# Non‐Pharmacological Interventions for People With Dementia Who Live Alone: A Systematic Review

**DOI:** 10.1002/gps.70059

**Published:** 2025-02-26

**Authors:** Sarah Polack, Georgia Bell, Barbora Silarova, Molly Hebditch, Alison Tingle, Andrew Sommerlad, Elena Portacolone, Kath Sykes, Naji Tabet

**Affiliations:** ^1^ Centre for Dementia Studies Brighton and Sussex Medical School Brighton UK; ^2^ Centre for Health Services Studies University of Kent Canterbury UK; ^3^ Centre for Research in Public Health and Community Care University of Hertfordshire Hatfield UK; ^4^ Division of Psychiatry University College London London UK; ^5^ Camden and Islington NHS Foundation Trust London UK; ^6^ Institute for Health & Aging (EP) University of California San Francisco San Francisco California USA; ^7^ NIHR Applied Research Collaboration Kent Surrey & Sussex/ Health Innovation Kent Surrey Sussex Beehive Gatwick UK

**Keywords:** dementia, interventions, living alone

## Abstract

**Objectives:**

Approximately one third of people with dementia live on their own and they face an increased risk of unmet needs and loneliness. This systematic review aimed to identify and describe non‐pharmacological interventions that have been evaluated for people with dementia living alone and to examine the effectiveness of these interventions.

**Methods:**

Following PRISMA guidelines, six databases were systematically searched: MEDLINE, Embase, CINAHL, PsycINFO, Social Care online, and ClinicalTrials.gov. Studies that reported on the impact or experience of an intervention for people with dementia living alone in the community (not long‐term care) and that had been published since 2000 were included in the review. No restrictions were applied in terms of study design or outcome measures. Study risk of bias was assessed, and a narrative approach was used to synthesize findings.

**Results:**

Thirteen studies of 13 different interventions were included, grouped into five intervention categories: home‐based dementia case/care management (*n* = 4), technology (*n* = 3), social (*n* = 3), cognitive (*n* = 2) and psychological (*n* = 1). There was one randomized controlled trial (RCT), and two economic evaluations that used data from RCTs. Most other studies were small‐scale, and only two were evaluated to have low risk of bias. Most studies reported positive or mixed findings in terms of the intervention's impact on the person with dementia or aspects of feasibility. However, studies were heterogeneous in terms of intervention, study design, and outcomes.

**Conclusions:**

This review of a limited body of research highlights the potential for interventions to support people with dementia who live alone. It also identifies key evidence gaps and the need for more robust and comparable research to better understand what works, why, for who, and how. Involving people with dementia who live alone in the design, implementation, and evaluation of these interventions will be crucial to ensure that their needs and preferences are met.

**Summary**

- Many people with dementia (approximately 30%) live on their own; however, they are often underserved by dementia policy, practice, and research, especially with regard to interventions.
- This systematic review identified 13 non‐pharmacological interventions evaluated for people with dementia who live alone. These interventions were grouped into five categories: home‐based dementia case/care management (*n* = 4), technology (*n* = 3), social (*n* = 3), cognitive (*n* = 2), and psychological (*n* = 1).
- Findings highlight promising results related to home‐based dementia care management and interventions that promote social interaction. However, conclusions on effectiveness are limited due to the limited number of robust studies and the heterogeneity in intervention, study design, and outcomes.
- There is a noticeable absence of people with dementia living alone in the design, implementation and evaluation of interventions. Another gap relates to evidence specifically for people with dementia who live alone and have no family or, other unpaid, carer support. These gaps need to be addressed to better inform new innovations and evidence‐based practice for this population.

## 1 Introduction

There are than 55 million people with dementia, globally, with projections forecasting that this number will exceed 150 million people by 2050 [1]. A substantial proportion of people with dementia live on their own; estimates range from 20% to 50% in Europe and North America [2, 3, 4, 5]. Despite the high number of people with dementia living alone, dementia‐related policy, practice, and research are often tailored to dyads—namely, the person with dementia and a cohabiting care‐partner (usually a spouse, partner, or adult child). As a result, people who live alone risk falling through the gap [6, 7]. For example, they may be excluded from dementia research (e.g., clinical trials) or services when the presence of a care‐partner is required; interventions may be designed and optimized for dyads, such as requiring a partner to provide care or to reinforce learning from a psychosocial intervention [3, 6]. However, this situation is gradually shifting, and there is a small, but growing body of research exploring the characteristics, experiences, and needs of people with dementia who live alone [2, 6, 7, 8, 9, 10, 11, 12].

The International Classification of Functioning (ICF), a biopsychosocial model of functioning and disability [13], describes that a person's participation and activities are influenced by their health condition/impairment (e.g., dementia) *in interaction* with contextual factors at the individual and environmental level. For example, living alone is often used in studies as a proxy for social isolation [14]; however, with appropriate support and enabling environments, some people with dementia living alone can—and do—live socially active, connected lives [2, 8, 15]. Some people with dementia prefer living alone rather than living with a family member or in residential care settings due to advantages such as the ability to remain in their home environment, lower costs of existing accommodations, freedom, autonomy, and the ability to maintain social networks [10, 15].

People with dementia live alone for different reasons and in different circumstances. For example, some live alone with relatives or friends nearby whereas others lack any informal support. Their experiences are also shaped by intersecting social identities (e.g., ethnicity, race, sexuality, gender). People with dementia living alone are, therefore, a diverse group. However, they also often share commonalities in terms of additional needs, risks, and barriers related to living alone. Such challenges include greater difficulty identifying and accessing support, particularly for people with no or limited contact with family member(s) to help them advocate for, navigate, or travel to support services or access help during an emergency [6]. In addition, people with dementia living alone are more likely to have unmet needs, particularly related to self‐care, nutrition, home upkeep, safety, and medication management than those who live with others [4, 10, 12, 16], and changing needs over time are less likely to be detected [17]. On average, people with dementia living alone are at greater risk of social isolation [12, 18] and loneliness [2, 11].

Research on the experiences and characteristics of people with dementia living alone typically points to a need for tailored support, services, and interventions to address their specific needs [2, 7, 8]. However, synthesized evidence on interventions and support that have been evaluated for people with dementia who live alone is lacking. Such information is needed to inform policymakers and practitioners on how best to enable and support people with dementia who live on their own and to guide future research priorities in this area. The objectives of the current systematic review are to (i) identify and describe non‐pharmacological interventions that have been evaluated to support people with dementia living alone and (ii) examine the feasibility, acceptability, and effectiveness of these interventions.

## 2 Materials and Methods

We undertook a systematic review of studies reporting on non‐pharmacological interventions for people with dementia who live alone. The review was registered with PROSPERO (CRD42023491618) and reported according to PRISMA guidelines [19] (See PRISMA checklist in Supporting Information S1).

### 2.1 Eligibility Criteria

We used broad inclusion criteria on study design, intervention type, and outcomes because of the anticipated limited research in this area.

Inclusion criteria: We did not apply restrictions on study type or outcome measure; any qualitative, quantitative, or mixed‐methods study, with or without a control or comparison group, that reported on the feasibility, experience, or effectiveness of an intervention was eligible. We included any non‐pharmacological interventions specifically targeting people with dementia living alone and those aimed at people with dementia in general, as long as results for people living alone were presented separately. Studies were included if they reported findings for people with dementia, of any subtype or severity, who were living alone in the community. Studies that included participants with a range of living arrangements (e.g., some living alone and some with family members) were included if the findings for people living alone were presented separately. We included studies that reported on outcomes or experiences for paid carers or family members (who lived elsewhere) as long as findings relating to the person with dementia were also presented.

Exclusion criteria: Papers that only described an intervention (e.g., study protocols or descriptions of intervention developments) but did not use research methods to assess feasibility, acceptability, or effectiveness were excluded. We also excluded studies conducted in long‐term care facilities, institutional settings, or supported accommodation.

### 2.2 Search Strategy and Selection Criteria

We conducted systematic searches of six databases (i.e., MEDLINE, Embase, CINAHL, PsycINFO, Social Care online and ClinicalTrials.gov) on February 15, 2024. We used search terms related to “dementia” AND “living alone” OR “social isolation” (See Supporting Information S3 for full search string). We conducted reference tracing of all included papers and relevant systematic reviews. Our searches were restricted to articles published in English and since 2000. Conference abstracts were included if sufficient detail was provided, or we were able to contact the authors for further details. We did not include theses/dissertations or grey literature.

### 2.3 Screening and Data Extraction

Titles and abstracts were independently screened by two reviewers; all articles were screened by SP and one of four other reviewers (GB, BS, MH, AT). Articles considered potentially relevant underwent a full text review by SP and GB. Discrepancies were discussed to reach consensus.

Data were extracted by SP, and checked by GB, into a standardized Excel form. The extracted data included publication details (author, year of publication, title, country), aims/objectives, methods (study design), participant characteristics (number, age, sex, type/severity of dementia, living situation), intervention characteristics (type, delivery mechanisms, intervention components, duration, use of theory, and co‐design), and outcomes (type, findings; narrative and statistical). We extracted all compatible results for quantitative outcomes, including the effect measure(s) provided in the articles. For example, if two different quality of life (QoL) measures were used, findings for both were recorded, and we recorded both primary and secondary outcomes and results from unadjusted and adjusted analyses. For two conference abstracts, we successfully contacted the author to gather relevant information.

### 2.4 Quality Assessment and Risk of Bias

We used the Joanna Briggs Institute critical appraisal tools [20]. SP and GB independently assessed the reviewed studies and categorized them as having low risk of bias (all or almost all of the criteria were fulfilled and those that were not fulfilled were considered unlikely to alter the conclusions of the study), medium risk (some of the criteria were fulfilled, and those not fulfilled were thought unlikely to alter the conclusions of the study), or high risk (few or no criteria were fulfilled, and the conclusions of the study were considered likely to change with their inclusion). We did not exclude studies based on their risk of bias.

### 2.5 Narrative Synthesis

We conducted a narrative synthesis which is suitable for reviews including studies that are heterogeneous in terms of design, interventions and outcomes. Following Popay et al. [21], we iteratively moved among their four recommended steps: (i) developing a theory of how interventions work, considering why and for whom (completed prior to the review to guide the questions and approach); (ii) developing a preliminary synthesis of included studies; (iii) exploring relationships in the data (e.g., we created figures to provide a summary overview of the relationship between the results and the intervention type and outcome measures); and (iv) assessing the robustness of the synthesis product. To aid interpretation, we summarized studies as positive (positive impact reported, either statistically significant improvement in all quantitative outcome measures or qualitatively assessed), null (no significant change), negative, or mixed (findings were a mix of positive and null). Meta‐analyses were not possible due to study heterogeneity. For ease of interpretation tables are structured by intervention category, within which quantitative studies are presented first.

## 3 Results

**FIGURE 1:**
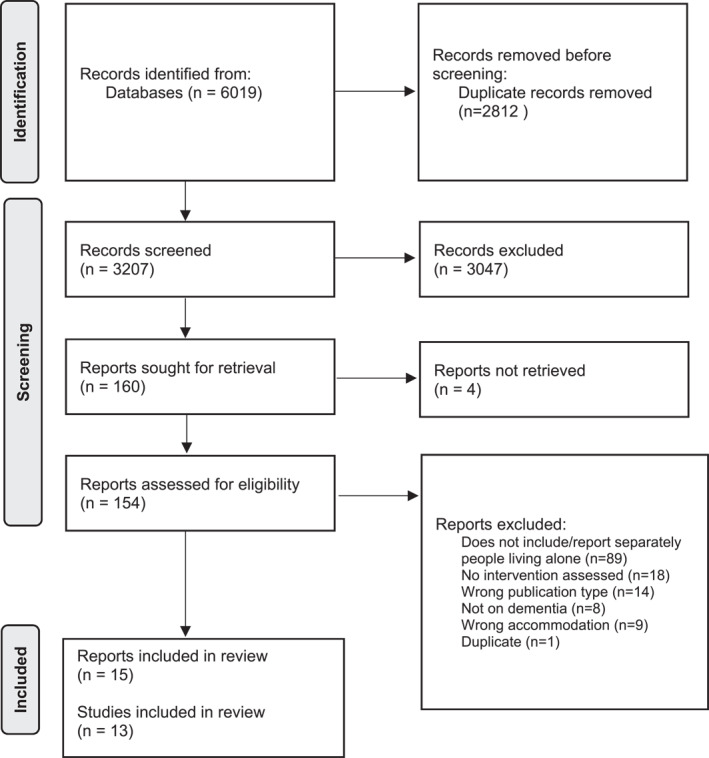
PRISMA flow diagram.

We identified 6019 records through database searches (Figure 1). After removing 2812 duplicates, 3207 articles were retained for title and abstract screening and 160 full texts were reviewed (see Supporting Information S2), of which 15 met our inclusion criteria. Three articles were linked to the same study which was a cluster randomized controlled trial (RCT); one presented the RCT results after a 12‐month follow‐up while the other two reported on similar economic analyses using data from the RCT after a 24‐month follow‐up. Therefore, in total, there were 13 studies of 13 interventions from the 15 articles included in the review.

### 3.1 Study Characteristics

All studies were conducted in high‐income countries (Table 1). The number of studies conducted has increased over time, from only one published between 2001 and 2010 to seven between 2011 and 2020 and five since 2020. Of the seven studies that reported on condition severity, people with mild or moderate dementia (*n* = 5) were most frequently included. Before and after (intervention) studies were the most common study type (*n* = 6); only one of these included a control group. There was one RCT. Three studies used qualitative methods, and there were two economic evaluations conducted using data from RCTs.

**TABLE 1:** Study characteristics (*n* = 13).

Characteristic	*N* ^a^	%
Region		
Asia	4	31%
Europe	6	46%
North America	3	23%
Country		
United Kingdom	5	38%
USA	4	31%
Germany	1	8%
Japan	2	15%
South Korea	1	8%
Publication decade		
2001–2010	1	8%
2010–2020	7	54%
2021–2024	5	38%
Participant focus		
People with dementia living alone, only	9	69%
People with dementia, including people living alone (sub‐group analysis)	4	31%
Study design		
RCT	1	8%
Economic evaluation, within randomised controlled trial	2^a^	15%
Before‐after, with control	1	8%
Before‐after, no control	5	38%
Mixed methods, post‐intervention only	1	8%
Qualitative	3	23%
Single case study	1	8%
Intervention type		
Home‐based dementia care management	4	31%
Technology	3	23%
Social	3	23%
Cognitive	2	15%
Psychological	1	8%
Intervention delivery		
Home‐based, one‐to‐one	11	85%
Community/online group	2	15%
Risk of bias		
Low	2	15%
Medium	6	46%
High	6	46%

^a^ One economic evaluation was conducted within the RCT included in this review, hence total equals > 100%.

Most interventions specifically targeted people with dementia who live alone (*n* = 8). Four included general community‐dwelling people with dementia with sub‐group analyses of those living alone.

### 3.2 Risk of Bias

Five quantitative or mixed methods studies were judged as high risk of bias and four as medium. Common methodological limitations were lack of control groups, lack of information about the reliability of outcome measurements and statistical issues (e.g., small sample sizes, absence of power calculations, descriptive analysis only, or inappropriate statistical methods). However, it should be noted that three of these were explicitly pilot or feasibility studies. One economic evaluation, using data from an RCT, was judged as low risk of bias, although it included a sub‐group analysis for people living alone with no power calculation for this group. Of the qualitative studies, two were assigned as medium risk (limitations included lack of reflection on researcher positionality and conceptual or theoretical considerations) and one as low risk.

### 3.3 Relationships Within and Among Studies

#### 3.3.1 Intervention Type

**FIGURE 2:**
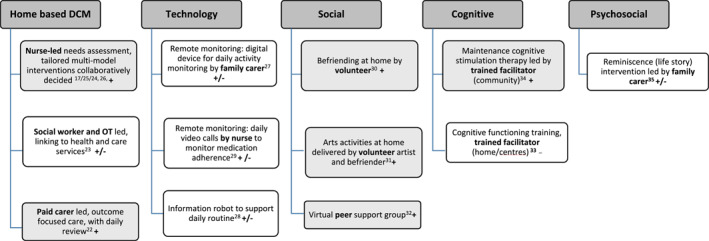
Summary of interventions, delivery agent and impact. Key: + (shaded grey) ‘positive’ impact (positive impact reported, either statistically significant improvement in all quantitative outcomes measures, or qualitatively assessed); **−** ‘no change’ (no evidence of significant change); **+/−** ‘mixed’ (findings were a mix of positive and no change). The superscript numbers correspond to the numbered references listed at the end of this article. DCM: Dementia Care Management; OT: Occupational Therapist.

Most studies described the intervention approach and rationale in reasonable detail, although descriptions of how interventions were developed, or theoretical underpinnings were generally lacking. We classified interventions into five broad categories (Figure 2): dementia care management (DCM) [17, 22, 23, 24, 25, 26], technology/digital [27, 28, 29], social [30, 31, 32], cognitive [33, 34], and psychosocial [35]. Interventions were most frequently delivered by nurses [17, 24, 25, 26, 29] or other healthcare and care professionals [22, 23, 28, 33, 34], followed by family carers living elsewhere [27, 35] and trained facilitators and volunteers [30, 31]. All interventions except one were home‐based [33]. Six studies explicitly involved family caregivers living elsewhere as part of the intervention [22, 24, 27, 35] and/or as study participants [22, 24, 26, 27, 29, 35].

All four DCM interventions involved regular (weekly–monthly) home visits to the person with dementia, with the duration varying from 12 weeks to 12 months. Two (one in Germany, one in Japan) were led by dementia‐specific trained nurses and involved assessments and tailored care plans developed and implemented in collaboration with other health professionals (e.g., GPs) [17, 24, 25, 26]. One, in the US, involved weekly home visits by a social worker with the aim of increasing the utilization of health and care support [23] and the other was ‘outcome‐focused’ care led by a paid carer in the UK, with outcomes jointly agreed upon between the person with dementia and family members and then reviewed daily [22].

Three different technology‐based interventions were studied, but they all supported aspects of daily routine for the person living alone, including (i) a device plugged into a regularly used home appliance (e.g., kettle) to enable remote daily routine monitoring by a family member (UK) [27]; (ii) daily video calls by a nurse to monitor medication compliance (US) [29]; and (iii) an information robot to support daily routines (Japan) [28].

Three social interventions were identified, including befriending by trained volunteers in Scotland [30], weekly arts‐based activities in England facilitated by an artist and befriender [31], and a weekly virtual peer support group in the US [32].

Two studies explored interventions focused on cognitive functioning [33], including an extended (16–24 weeks) group‐based cognitive stimulation therapy in the UK [33] and cognitive functioning training (including social contact and activities) delivered at home or in care centers in Japan [34]. The psychosocial intervention as part of a study conducted in the US focused on life review, a reminiscence activity provided virtually (due to the COVID‐19 pandemic) by a family member [22].

### 3.4 Outcomes

**FIGURE 3:**
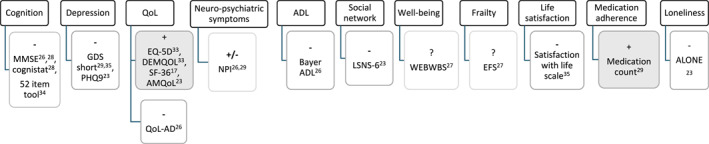
Outcome measures used in the included studies. Key: + (shaded grey) ‘positive’ impact (statistically significant improvement in all quantitative outcomes measures); − ‘no change’ (no evidence of significant change); **+/−** ‘mixed’ (findings were a mix of positive and no change); ? ‘Unclear’. The superscript numbers correspond to the numbered references listed at the end of this article. ADL: Activities of Daily Living; ALONE: ALONE scale; AMQoL: Aging Mobility and Quality of Life Survey; Bayer ADL: Bayer Activities of Daily Living Scale; Cognistat: cognitive screening and assessment tool; DEMQOL: Dementia quality of life; EFS: Edmonton Frail Scale; EQ‐5D: Euroqol 5‐Dimension; GDS: Geriatric depression Scale; LSNS‐6: Lubben Social Network Scale; MMSE: Mini‐Mental State Examination; NPI: Neuropsychiatric Inventory; PHQ‐9: Patient Health Questionnaire 9 item; QoL‐AD: quality of life in Alzheimer's Disease; SF‐36: 36 item short form survey; WEBWBS: The Warwick‐Edinburgh Mental Wellbeing Scale.

Multiple outcome domains, using different patient‐reported outcome measures (PROMs), were used within and across the nine quantitative and mixed‐method studies (Figure 3, Table 2). No clear patterns emerged in the intervention type and outcome measures used. Most studies used pre‐existing validated PROMs, including both condition/age specific (e.g. DEMQoL) and generic (Luben Social Network Scale, LSNS‐6) tools, though it was unclear whether the generic PROMs had been validated for use with people with dementia. The most frequently assessed outcomes were QoL (four studies, using five different instruments) and cognition (four studies, using MMSE and a South Korean national assessment tool); three studies measured depression, and two assessed activities of daily living and neuropsychiatric symptoms. Social networks, safety, frailty, well‐being, and satisfaction with life were each assessed in only one study. Family/caregiver‐reported outcomes were measured in four studies, most commonly in regard to caregiver burden and well‐being, while satisfaction with life and positive aspects of caregiving were each assessed in one study. Four studies involved qualitative interviews or open‐ended questions to explore aspects of acceptability, feasibility, and experience of the intervention. However, in two of these studies, only a family member—not the person with dementia living alone—was interviewed.

**TABLE 2:** Details of study methodology, by intervention domain.

First author, year, country	Study aim	Study design	Target group	Participant demographics (refers to people with dementia living alone, unless specified otherwise)	Outcome assessment measures	Outcome assessment measures
					Person with dementia	Family carer (FC)
Home‐based dementia care management (DCM)						
Thyrian et al. 2017 [26] Germany^a^	To assess the effectiveness of collaborative DCM (12 month follow‐up)	Cluster RCT	People with dementia; sub‐group analysis for living alone	12 month follow‐up: Intervention: 291 (151 LA) Control ‐ CAU: 116 (53 LA) Females: 60%^b^ Screen positive using DemTect (mild‐severe) Age: ≥ 70 years	QoL (QoL‐AD), Cognition (MMSE) ADL (Bayer ADL) Behavioural symptoms (NPI) Medication: Use of antidementia drugs,	Caregiver burden (BICS‐D)
Radke et al. 2020 [17] & Michalowski et al. 2019 [25] Germany^a^	To identify socio‐economic and clinical sub‐groups that benefit most from collaborative DCM	Cost‐effectiveness analysis, within cluster RCT	People with dementia; sub‐group analysis for living alone	Analysis included participants who completed baseline and at least one follow‐up (12 or 24 month) assessment or died: Intervention: *N* = 315 (163 LA) Control, CAU: *N* = 129 (61 LA) Females: 60%^b^ Screen positive using DemTect (mild‐severe) Age: ≥ 70 years	HrQoL (SF‐12), Utilities, QALYs, ICERs. Published unit costs. Healthcare resource utilization (interviews).	
Gibson et al. 2023 [23] USA	Pilot study of case management services programme: Services to age in Your home (STAY home)	Pre‐post, no control group (Pilot/proof of concept study)	People with dementia living alone	Total: 12 (8 female) Age (mean): 70	Service use frequency, Depression (PHQ9), Social network (LSNS‐6), Loneliness (ALONE), Activities of daily living, Safety assessment checklist, Quality of life (AMQoL);	
Gethin‐Jones 2014 [22], UK	Explore impact of outcome‐focused homecare on well‐being of family carers and perceived of impact on relative with dementia who lives alone.	Qualitative;	People with dementia living alone	Total: 20 family carers (FC)		Semi‐structured interviews concerns, perception of intervention and perceived impact on person with dementia. Single, author written, question (with likert scale) on well‐being of themselves and person with (dementia)
Kitamura et al. 2019 [24] Japan	Explore caregiver experiences of home‐visit nursing for people with dementia who live alone	Qualitative,	People with dementia living alone	Total: 5 (4 female) Age range: 78–88		Interviews on experiences of caregiving before and during the intervention; perceived impact on person with dementia
Technology						
Smith G et al. 2007 [29] USA	To assess impact of tele‐video monitoring on medication self‐administration and mood for people with dementia living alone	Before‐after, no separate control group	People with dementia living alone	Total: 14 Mild dementia Mean age > 79 years	Medication counts Behavioural symptoms (NPI) Depression (GDS short). Open questions about experiences of intervention	Open‐ended questions about experiences of intervention
Fowler‐Davis et al. 2020 [27] UK	Pilot study exploring impact and feasibility of digital monitoring device on well‐being of person with dementia and family carer	Before‐after, no control (mixed methods)	People with dementia living alone	Total: 30 dyads: Person with dementia living alone (23 female) + relative dyads Age range: 65–96	Well‐being (WEBWBS short) Frailty (EFS)	Well‐being (WEBWBS short) Burden (ZBI) Semi‐structured interviews on experience of using device Digital monitoring data
Mizuno 2021 [28] Japan	Assess effect of information support robots on agitation in people with Alzheimer's who live alone	Single case study	People with dementia living alone	1 woman, Age: 70 Alzheimer's disease	Cognition: MMSE‐J and COGNISTAT Activity data: restless episodes using remote monitoring	
Social						
Gibson, 2017 [32] USA	Explore challenges and opportunities of virtual support group for persons living alone with dementia,	Mixed methods, post intervention	People with dementia living alone	Total: 12 (7 female) Within 2 years of diagnosis; mean age 66, 58% female	Survey developed by author about developing end of life plan and access to services	
Eades et al. 2018 [31] UK	To evaluate an arts based outreach programme for people with dementia living alone	Qualitative	People with dementia living alone	Total: 6 (3 female) Mild/moderate dementia, Ages 70–90, 4 artists, 3 befrienders	Qualitative interviews with topic guide	
Andrew et al. 2022 [30] UK	Explore how people living alone with dementia experienced befriending	Qualitative	People with dementia living alone	Total: 3 people (2 female); different types of dementia, age range: 69–90;	Qualitative case study—5 unstructured conversations (around befriending, everyday life, social networks and biography) and ethnographic observations.	
Cognitive						
Brown et al. 2019 [33], UK	Assess cost effectiveness of maintenance cognitive stimulation therapy: Examining influence of cognitive ability and living arrangements	Cost‐effectiveness analysis, within RCT	People with dementia; sub‐group analysis for living alone	Intervention: 123 (23 LA); control (CAU): 113 (23 LA) > 60% female^b^ Mild‐moderate dementia	Cost (various sources), HRQoL (EQ‐5D and DEMQOL), QALYs, CER	
Ju et al. 2019 [34]; Japan	To evaluate the national long‐term care insurance‐funded cognitive functioning programme for people with mild dementia	Before‐after, with control	People with dementia; sub‐group analysis for living alone	Intervention: 352 (80 LA) Control 1: 337 (185 LA), control 2 1615 (378 LA) Mild dementia, hospitalised for behavioural or psychological symptoms Age: > 70^b^	Cognitive function: 52 item “national care need‐assessment tool”	
Psychological						
Miyawaki et al. 2023 [35]; USA	Explore feasibility and efficacy of ‘caregiver Provided life review (C‐PLR)' a carer delivered depression intervention for people with dementia and mild depression. Delivered virtually due to COVID	Before‐after, no control	People with dementia; sub‐group analysis for living alone	Total: 25 dyads (12 LA) Age: > 70 years^b^ 84% female^b^	Depression (GDS‐SF); Satisfaction with life scale.	Satisfaction with life scale. Burden (BSFC‐S) Caregiving (PAC) scale; Relationship quality

Abbreviations: AMQoL: Aging Mobility and Quality of Life Survey; Bayer ADL: Bayer Activities of Daily Living Scale; BICS‐D: Berlin Inventory of Caregiver Stress—Dementia (BICS‐D); BSFC: Burden Scale for Family Carers—short; CAU: care as usual; Cognistat: cognitive screening and assessment tool; DEMQOL: Dementia Quality of life; EFS: Edmonton Frail Scale; ALONE: ALONE scale; EQ‐5D: Euroqol 5‐Dimension; FC: family carer; GDS: Geriatric depression Scale; HrQoL: Health related quality of life; ICER: Incremental Cost Effectiveness Ratios; LA: Person with dementia living alone; LSNS‐6: Lubben Social Network Scale; MMSE: Mini‐Mental State Examination; NPI: Neuropsychiatric Inventory; PAC: Positive Aspects of Caregiving; PHQ‐9: Patient Health Questionnaire 9 item; QALYs: Quality Adjusted Life Years; QoL‐AD Quality of Life in Alzheimer's Disease; RCT: Randomised Controlled Trial; SF‐36: 36 item short form survey; WEBWBS: The Warwick‐Edinburgh Mental Wellbeing Scale; ZBI: Zarit Burden Interview.

^a^ These articles are part of the same overall study but are presented as two separate rows in order to describe the different sub‐study aims and analyses.

^b^ Numbers provided for all participants only, not sub‐group living alone.

### 3.5 Evidence of Acceptability, Feasibility, and Effectiveness

All studies except one [34] reported a positive effect on at least one quantitative outcome or aspect of impact or acceptability that was explored qualitatively. However, findings were often mixed with improvement in some (but not all) outcome measures (Figure 3, Table 3).

**TABLE 3:** Summary of key study findings by intervention domain.

First author, year, country	Study design and intervention summary	Participant numbers (people with dementia living alone unless specified otherwise)	Key findings for people with dementia living alone	Risk of bias
Home‐based dementia care management				
Thyrian et al. 2017 [26] Germany^a^	Cluster RCT; Collaborative DCM model, nurse led	Intervention: 151 Control ‐ CAU: 53	For people living alone, statistically significant reduction in behavioural and psychological symptoms; no statistically significant impact on any other outcomes. **Mixed** **^b^**	Medium
Radke et al. 2020 [17] & Michalowski et al. 2019 [25] Germany^a^	Cost‐effectiveness analysis, within cluster RCT; Collaborative DCM model, nurse led	Intervention: 163 Control, CAU: 61	For people living alone incremental costs were lower and QALYs higher (stronger dominance). Probability of cost‐effectiveness was higher for those living alone versus people living with others. **Positive** **^b^**	Low
Gibson et al. 2023 [23] USA	Pre‐post, no control group (Pilot/proof of concept study) Social worker and OT led, linking to health and care services	12	Intervention was feasible: high adherence to weekly visits (75%). Significant increases in involvement with health and social service providers after intervention. QoL increased from pre‐ to post‐intervention. Unclear for other outcomes. **Mixed** **^b^**	High
Gethin‐Jones 2014 [22], UK	Mixed methods; Paid carer led, outcome focused care.	20 family carers (FC)	Quantitative; caregiver perceived well‐being of person with dementia improved from ‘poor’/’as bad as it gets' to ‘neither good or bad’ for 17 of the 20 participants Qualitative: Some improvement in subjective well‐being of family carer attributed to consistency in care (i.e. provided by fewer different people); feeling more supported, less isolated, improved communication. Family carer (FC) perceived some improvements for person with dementia (although limited data): They appeared more settled, less agitated and less likely to reach crises. **Positive** **^b^**	High
Kitamura et al. 2019 [24] Japan	Qualitative; Nurse led DCM	5	Qualitative: Family carer (FC) reported increased feelings of security, reduction in stress, deeper understanding of dementia and appropriate care. FC perceived some improvements in symptoms and QoL for person with dementia (data very limited). **Positive (family carer only)** **^b^**	Medium
Technology				
Smith et al. 2007 [29] USA	Before‐after, no control (mixed methods). Daily video calls by nurse to monitor medication adherence	14	Medication compliance more stable and higher (81%) at endline compared to control (62%, *p* < 0.05) suggesting intervention may prevent deterioration in compliance. Decline/no change in depression, cognition or behavioural symptoms. Intervention was feasible. FC reported monitoring was reassuring and beneficial for medication compliance, social support/rapport for person with dementia (with nurse assistant) and prevented relocation. Some (10%–15%) contacts were missed usually because person with dementia was engaged in other activities. Some found intervention intrusive, but limited exploration of this. **Mixed** **^b^**	High
Fowler‐Davis et al. 2020 [27] UK	Before‐after, no control (mixed methods; pilot study); Digital device for daily activity monitoring by family carer	30 dyads (person with dementia living alone + family member)	Mixed results and descriptive statistics only. Person with dementia: Out of 30 participants, 13 improved, 17 declined in well‐being, 7 improved, 13 declined, 10 stayed the same in frailty. FC: 10 improved, 5 stayed same 15 declined wellbeing; 18 improved, 10 declined, 2 same in caregiver burden Device demonstrated stability of daily routine. Device reported as acceptable, although limited empirical evidence; FC reported it provided reassurance, promoted less checking and therefore enhanced social contact with their family member with dementia and that person with dementia living alone appreciated being ‘connected’ to FC. **Mixed** **^b^**	High
Mizuno 2021 [28] Japan	Single case study; Information robot to support daily routine	1	Cognition: Small (4‐points) increase in MMSE‐J score and 3 areas of COGNISTAT (naming, constructional ability, and similarities) but they remained at impaired level. Restlessness: Varied by type; reduction in some, no change/increase in others. **Mixed** **^b^**	Medium
Social				
Gibson 2017 [32] USA	Mixed methods, post intervention (pilot study); Virtual peer support group	12	After the intervention the majority (*n* = 10) were willing to develop an advanced care plan and identify someone to act as their care partner. Results suggested increased understanding about dementia and feelings of social support **Positive** **^b^**	High
Eades et al. 2018 [31] UK	Qualitative Arts activities at home	6	Good engagement with arts activities; stimulated reminiscence, sharing memories/stories, areas of shared interest and meaningful connections Positively challenged preconception, stigma and fear about dementia among befrienders and artist. Unclear how much of positive impact was due to art activity or to the social interaction. **Positive** **^b^**	Medium
Andrew et al. 2022 [30] UK	Qualitative; Befriending	3	Facilitated valuable, authentic and long‐lasting friendship with relational quality and equality (mutual exchange), trust. This was particularly important in context of altered and reduced contact with friends and family. Helped remove environmental barriers (e.g. transport) to support, social inclusion and agency. Memory loss impacted ability to recall past or upcoming befriending visits which could cause uncertainty or worry, some confusion about who the befriender was and fear of abandonment. Regular, face‐to‐face visits and from same person and memory aids seemed to help. **Positive** **^b^**	Low
Cognitive				
Brown et al. 2019 [33], UK	Cost‐effectiveness analysis, within RCT; Maintenance cognitive stimulation therapy	Intervention: 23 Control (CAU0): 23	For person living alone maintenance cognitive stimulation therapy (CST) dominates (i.e. was more cost‐effective) or had modest incremental cost per unit increase in HRQoL, in contrast to other sub‐groups, where standard length CST, followed by usual care, dominates. **Positive** **^b^**	Medium
Ju et al. 2019 [34]; Japan	Before‐after, with control; Cognitive functioning training	Intervention0: 80 Control 1: 185 Control 2: 387	In the full study sample less cognitive decline in intervention group compared to control groups. Among PLA, however, there was no significant change in cognitive function in the intervention group. **No change** **^b^**	Medium
Psychological				
Miyawaki et al. 2023 [35]; USA	Before‐after, no control; Reminiscence (life story) activity, delivered remotely by family	25 dyads (12 PLA)	Depression symptoms reduced overall and for people living with others, but this reduction was not statistically significant for people living alone after bonferonni correction. No significant changes in any of the other outcomes overall, or separately for PLA. Intervention feasible with good adherence and fidelity and did not increase FC burden; but feasibility was not reported on separately for people living alone. **Mixed** **^b^**	Medium

Abbreviations: CAU: care as usual; Cognistat: cognitive screening and assessment tool; FC: family carer; HrQoL: Health related quality of life; ICER: Incremental Cost Effectiveness Ratios; MMSE: Mini‐Mental State Examination; PLA: Person with dementia living alone; QALYs: Quality Adjusted Life Years; RCT: randomised control trial.

^a^ These articles are part of the same overall study but are presented as two separate rows in order to describe the results of the different sub‐studies.

^b^ ‘positive’ impact: positive impact reported, either statistically significant improvement in all (quantitative outcomes measures, or qualitatively assessed); ‘no change’: no evidence of significant change; ‘mixed’: findings were a mix of positive and no change.

QoL improved in three of the four studies that measured it, while there was no significant change in cognition in the four studies that measured this. Interpreting change in the context of dementia is complex; for example, no change in cognitive function over time can be interpreted positively in the context of a progressive disease.

Overall, there was promising evidence from studies evaluating DCM (Table 3). A cluster‐RCT in Germany, of nurse‐led DCM, found significant reduction in the neuropsychiatric symptoms of dementia for people living alone, but no change in QoL after 12 months. This intervention was particularly cost‐effective for people with dementia living alone compared to those living with others [17, 25]. A pilot study found that a DCM approach, led by social workers and occupational therapists, was feasible (high adherence to weekly visits) and indicated improvements in QoL scores and health and care utilization, but not secondary outcome measures (e.g., depression, loneliness). However, the study was small (*n* = 12) and not powered to assess change in these outcomes [23]. In two qualitative evaluations of DCM, family caregivers (living elsewhere) felt better supported and less stressed and had an improved understanding of dementia care; one of the studies largely attributed this to the consistency of care providers [22]. Some positive impacts for the person with dementia (e.g., reduction in agitation and reaching crises) were reported. However, these findings were limited and based on caregiver perceptions only [22].

Findings related to technology‐based interventions were mixed. Two remote monitoring interventions (a device allowing family members to monitor daily routines [27] and daily video calls by a nurse to check medication compliance [29]) were feasible (in terms of practicality and adherence [29] and acceptability to family members [27]) and provided reassurance for family members. For example, the use of a device to remotely monitor daily routines reportedly led to less checking and, therefore, better social contact with their relative with dementia. There were also perceived benefits for the person with dementia. For example, family members felt that nurses' daily video calls provided social support. However, there was very limited exploration of the acceptability or impact from the perspective of the person with dementia in either study. In terms of quantitative outcomes, the authors interpreted more stable medication compliance compared to the control phase, suggesting that daily calls from a nurse prevented deterioration. No clear changes in other quantitative outcomes for the person with dementia (depression, well‐being, frailty, cognition, or behavioral symptoms) were evident in either study, although the sample sizes were small (< 30 people). Some cognitive improvements and a reduction in restlessness after the use of an information robot were identified [28], although the findings were mixed and limited to one person only.

All three studies of social interventions reported positive impacts on the person with dementia. Benefits of befriending [30] and weekly home‐based creative activities [31]—explored through qualitative interviews with three and six people with dementia, respectively—included meaningful social connections, mutual exchanges, and sharing stories. Befriending also helped people with dementia living alone to overcome environmental barriers (e.g., transport) to social inclusion and improved agency. Challenges were also highlighted, such as confusion about who the befriender was and fear of abandonment, although these were reduced with regular face‐to‐face visits and the use of memory aids [30]. A small study of a virtual peer support group achieved its intended intervention goal of supporting people to develop advance care plans [32].

Findings related to cognitive interventions were mixed and limited to two studies using different outcome measures. A group‐based maintenance cognitive stimulation therapy intervention (i.e., extending cognitive stimulation therapy by 14–24 weeks) was suggested to be cost‐effective for people with dementia living alone, but not those living with others. However, the authors warrant caution in interpretation due to the small sample sizes [33]. In contrast, a national cognitive functioning training program in Japan was associated with less cognitive decline for people with dementia overall (compared to those not receiving the intervention), but not among people who lived alone; although there were limitations in terms of control group comparability as controls were selected from people who were not eligible for the cognitive training because they were considered either less (control Group 1) or more (control Group 2) ‘dependent’ than the intervention participants [34]. A single study of a psychosocial intervention using a remote caregiver‐delivered life review (based on reminiscence therapy) found that the intervention was feasible (adherence) and associated with a reduction in depression symptoms for people with dementia overall, but there was no statistically significant change in depression or life satisfaction for people who lived alone once Bonferroni adjustment was applied [35].

There were no clear patterns, across the studies, in how intervention effectiveness was related to participant's socio‐demographic and clinical characteristics (e.g. dementia severity) or study setting or context, although comparisons (within and between studies) were limited by a lack of variation (e.g., in age or dementia severity) among the participants and the lack of comparable study designs and outcomes.

## 4 Discussion

This review identified studies of 13 interventions evaluated for people with dementia who live alone; eight interventions explicitly focused on people living alone while five targeted general groups of people with dementia, with findings presented separately for those living alone. There were five intervention categories: home‐based DCM, technology, social, cognitive, and psychological. Most studies reported positive or mixed findings in terms of the impact of the intervention on the person with dementia and/or family member (living elsewhere) or aspects of feasibility. However, any robust conclusions are limited by the lack of high‐quality and comparable studies. One RCT [26] and two economic analyses using data from RCTs [17, 33, 35] were included, but they were not specifically designed or powered for people living alone. Overall, studies were mostly small scale and heterogeneous in terms of intervention, study design, and outcomes, limiting comparability and interpretation while also indicating the need for further development and testing of interventions for this group.

This review found promising evidence for DCM, which may be particularly appropriate for people with dementia who live alone, given the additional challenges they can face identifying, navigating, and physically accessing support [10]. In Germany, a nurse‐led collaborative DCM model was found to be particularly cost‐effective for people with dementia who lived alone compared to those living with others. High‐cost savings were attributed to reduced hospital and care‐home admissions [17, 35]. However, implementation barriers (e.g., high costs, shortage of nurses) have restricted the wide‐scale adoption of this model into routine practice [36]. The approach is complex, involving detailed needs assessments, regular home visits, and multi‐model interventions. It will be important to learn from implementation studies underway to better understand the mechanisms of impact [36]. The other DCM studies in our review also provided some evidence of positive impact, although conclusions are limited by study design variation and quality. Similarly, a Cochrane review indicated benefits of DCM for the general population with dementia, although evidence was mixed [37]. Given the considerable variation in DCM approaches in different contexts [38], there is a need for robust realist and process evaluations to better understand what works, for whom, and how.

Several studies in this review highlighted the potential benefit of interventions that promote social interactions and meaningful relationships, either directly through social interventions (e.g., befriending) or indirectly (e.g., regular nurse calls to check medication adherence). These findings are encouraging given concerns about social isolation and loneliness for people with dementia who live alone. However, it is difficult to draw strong conclusions about the nature of social interventions due to the lack of comparable studies and evidence gaps. For example, our review identified only two group‐based interventions, which is a notable gap considering that an umbrella review of interventions for people with dementia emphasized their potential importance in promoting social integration [39]. Attention must be paid to breaking down barriers to social interaction and participation, given that people living alone are more likely to face exclusion, either directly if services require the presence of a carer or indirectly due to, for example, a lack of transportation [6]. Interventions for people living alone may need to leverage existing non‐cohabiting social contacts or foster new networks through group interventions.

The potential for technology to support people living alone also warrants more attention. Digital solutions are increasingly viewed as an important component of the sustainability of health and social care systems. The three technology interventions in our review only focused on monitoring or supporting daily routines, giving limited attention to acceptability from the perspective of the person with dementia and related ethical implications. One small‐scale study (in the social category) pilot tested an online peer support group to encourage advance care planning [32]. There is growing, albeit still relatively limited, evidence for the benefits of digital interventions for general populations of people with dementia, including their effects on quality of life and social isolation [40]. In a recent review on digital access to health and social care services for people with dementia, participants in the included studies were predominantly family carers, and interventions were often dependent on the support from (usually younger) family members [41]. Considering the variations in digital access and the fact that dementia symptoms can make engaging with technology more difficult, these findings may not be generalizable to people living alone who have limited contact with family members. Therefore, co‐production and usability testing of technology solutions with people with dementia who live alone is needed.

This review highlighted substantial evidence gaps. Nearly half of the studies included a family member either in the intervention or as study participants. However, estimates suggest that up to 9% of people with dementia have no informal carer at all [6], and little is known about how these individuals can best be supported. Moreover, evidence on the acceptability of interventions other than drop‐out rates from the perspective of the person with dementia is lacking (e.g. satisfaction, preference, appropriateness of the intervention). In two studies, outcomes were reported by a family member only, not the person with dementia. Furthermore, there was no evidence that people with dementia were involved in the design or development of the interventions. There is an encouraging increase in the use of co‐design in dementia interventions and research [42], and this must extend to include to people who live alone.

In addition, we found no evidence of theoretical underpinnings for the identified interventions. Process evaluations for complex interventions, such as the collaborative DCM, were lacking. Such evaluations are needed to better understand pathways to impact and which components may or may not be contributing to success. Similarly, we found limited evidence on the role of individual (e.g., type and severity of dementia) or contextual (e.g., existing social networks, urban/rural location) factors in moderating the impact of interventions. Most studies had relatively small sample sizes and were lacking controls. Given that many dementia intervention studies do include people who live alone, we encourage more adequately powered sub‐group analyses as one way to expand evidence in this area. Few or none of the quantitative studies assessed outcomes related to social participation, loneliness, safety, life satisfaction, and food/nutrition, which is concerning considering that these outcomes have been reported to be worse for people living alone [2, 4, 12].

### 4.1 Strengths and Limitations

To our knowledge, this is the first systematic review of interventions to support people who live alone with dementia, providing a synthesiszed foundation to inform future research. We were deliberately broad in our search, given the expected lack of research in this area. Limitations include the inclusion of peer‐review articles in English language only, which may have missed relevant evidence published in grey literature and/or in other languages. We did not exclude studies based on their risk of bias; this limited the capacity for robust generalizable conclusions, but we considered it important to understand the state of the evidence in this area. Furthermore, we echo Rai et al.’s [40] call for better tools to critically appraise early stage studies (e.g., pilot or feasibility studies) as these are challenging to classify in terms of risk of bias, yet can contribute important learnings.

## 5 Conclusion

This review highlights a limited body of research on interventions to support people with dementia who live alone. Potential effectiveness of interventions, such as DCM and provision of social contact, is indicated but conclusions are limited by the lack of robust evidence and heterogeneity in interventions, study designs, and outcomes. Given the increasing number of people living alone with dementia, further robust larger‐scale research focused on interventions to support this population is urgently needed. Their involvement in the design and implementation of interventions will be crucial to ensure that their needs and preferences are appropriately met.

## Ethics Statement

The authors have nothing to report.

## Consent

The authors have nothing to report.

## Conflicts of Interest

The authors declare no conflicts of interest.

## Supporting information

Supporting Information S1

Supporting Information S2

Supporting Information S3

## Data Availability

The authors have nothing to report.
