# Supplementary material for: Non‐Pharmacological Interventions for People With Dementia Who Live Alone: A Systematic Review
Source: Int J Geriatr Psychiatry. 2025 Feb 26;40(3):e70059. doi: 10.1002/gps.70059 (PMC11864918; doi:10.1002/gps.70059)
Supplement: Supplementary file 2 — Supporting Information S2 [file GPS-40-e70059-s001.docx]

**Supplementary material 2 - Articles excluded articles based on full text review.**

| Reason for exclusion | Reference |
| --- | --- |
| Does not include/report separately people living alone (89) | (Anderson et al. 2022; Anonymous 2008; Bachle et al. 2018; Baker and Irving 2016; Begde et al. 2022; Behera et al. 2021; Boman et al. 2014; Bourne, Camic, and Crutch 2021; Brims and Oliver 2018; Brown, Cedar, and Tziraki 2022; Camic, Tischler, and Pearman 2014; Campbell et al. 2012; Cheng, Zhou, and Sabran 2024; Clark and et 2004; Curelaru et al. 2021; DePalma 2007; Derbring et al. 2023; Diaz Narvaez and Rughwani 2019; Domenicucci et al. 2022; Elliott and Gardner 2018; Enshaeifar et al. 2018; Femiola and Tilki 2017; Ferreira-Brito et al. 2021; Garrido et al. 2024; Gerritzen et al. 2020; Harris and Caporella 2014; Heins et al. 2021; Holthe et al. 2018; Holthe, Halvorsrud, and Lund 2022; Hum et al. 2020; Innes et al. 2021; Irazoki et al. 2020; Joshi et al. 2023; Kang et al. 2023; Karkou et al. 2023; Khosla et al. 2021; Kim et al. 2022; Kitamura et al. 2018; Koh, Ang, and Casey 2021; Lariviere et al. 2021; Lion, Szczesniak, et al. 2021; Lion, Szcześniak, et al. 2021; Livingston et al. 2014; Lloyd-Williams and et 2020; Löbe and AboJabel 2022; Lord et al. 2020; Luker et al. 2019; MacAndrew, Brooks, and Beattie 2019; Maffioletti et al. 2021; Maggio et al. 2023; Malmgren Fange et al. 2020; McGrattan et al. 2017; Mole et al. 2018; Moniz Cook et al. 2012; Moyle et al. 2017; Moyle, Murfield, and Lion 2021; Nourhashemi et al. 2008; Park et al. 2022; Pierce et al. 2017; Pinto-Bruno et al. 2017; Quail, Bolton, and Massey 2021; Rai et al. 2022; Russell, Hovey, and Fairlie 2005; Samtani, Stevens, and Brodaty 2021; Schneider et al. 2023; Scott et al. 2019; Semple, Willis, and de 2015; Shannon, Bail, and Neville 2019; Sharew 2022; Sharew et al. 2021; Sixsmith 2006; Starns, Karner, and Montgomery 2002; Streater et al. 2020; Struckmeyer and Pickens 2016; Sun 2018; Sun, Akhter, and Gabel 2022; Thomas et al. 2023; Tsuda et al. 2022; Tulliani et al. 2022; van der Roest et al. 2008; van Leersum et al. 2023; Van Mierlo et al. 2010; Villars et al. 2013; Watari et al. 2006; Wells et al. 2023; Wenborn et al. 2023; Wenborn et al. 2021; White 2016; Zhu et al. 2023; Zwierenberg et al. 2018) |
| No intervention assessed (18) | (Watari et al. 2006; Goodman-Casanova et al. 2020; Hagen et al. 2004; Manteau-Rao and Levenson 2018; Mulvenna et al. 2017; Nygård et al. 2020; Radke et al. 2020; Rahja et al. 2018; Research 2015; Rokstad et al. 2018; Salva et al. 2009; Shin et al. 2022; Souza 2017; Waal 2014; Waugh 2009; Wrede, Braakman-Jansen, and van Gemert-Pijnen 2021; Wubbeler et al. 2015; Schwartz 2013) |
| Wrong publication type (14) | (Costa and Doughty 2009; Foundations 2014; Greene, Ostroushko, and Melius 2022; Keogh et al. 2018; Koowattanataworn et al. 2022; Mourant 2014; Price 2007; Prick et al. 2011; Radke, Hoffmann, and Michalowsky 2019; Smith and Astell 2017; Xu et al. 2023; de las Heras and Burgos 2017; Kelly, Richardson, and Moss 2023; Neylon et al. 2017) |
| Not on dementia (8) | (Braspenning et al. 2022; Franco et al. 2008; Holloway 2022; Karp et al. 2008; Liang et al. 2022; Nygard, Starkhammar, and Lilja 2008; Windischbauer et al. 2023; Wu et al. 2023) |
| Wrong accommodation type (9) | (Dulaney et al. 2019; Inoue et al. 2012; Klemets, Maattala, and Hakala 2019; Koh et al. 2023; Kontos and et 2021; McGilton et al. 2012; Ridder and Aldridge 2005; Van Assche et al. 2024; Wilson 2015; O'Connor et al. 2009) |

**Reference list of excluded articles**

Anderson, M., R. Menon, K. Oak, and L. Allan. 2022. "The use of technology for social interaction by people with dementia: A scoping review." *PLOS Digital Health* 1 (6):e0000053. doi:<https://dx.doi.org/10.1371/journal.pdig.0000053>.

Anonymous. 2008. "Social isolation in community-dwelling seniors: an evidence-based analysis." *Ontario Health Technology Assessment Series* 8 (5):1-49. doi: doi:.

Bachle, M., S. Daurer, A. Judt, and T. Mettler. 2018. "Assistive technology for independent living with dementia: Stylized facts and research gaps." *Health Policy and Technology* 7:98-111. doi: doi:<https://dx.doi.org/10.1016/j.hlpt.2017.12.002>.

Baker, Keith, and Adele Irving. 2016. "Co-producing approaches to the management of dementia through social prescribing." *Social Policy and Administration* 50 (3):379-397. doi:.

Begde, A., M. Jain, E. Hogervorst, and T. Wilcockson. 2022. "Does physical exercise improve the capacity for independent living in people with dementia or mild cognitive impairment: an overview of systematic reviews and meta-analyses." *Aging & Mental Health* 26 (12):2317-2327. doi:<https://dx.doi.org/10.1080/13607863.2021.2019192>.

Behera, C. K., J. Condell, S. Dora, D. S. Gibson, and G. Leavey. 2021. "State-of-the-Art Sensors for Remote Care of People with Dementia during a Pandemic: A Systematic Review." *Sensors* 21 (14):08. doi:<https://dx.doi.org/10.3390/s21144688>.

Boman, Inga-Lill, Stefan Lundberg, Sofia Starkhammar, and Louise Nygård. 2014. "Exploring the usability of a videophone mock-up for persons with dementia and their significant others." *BMC Geriatrics* 14 (1):49-49. doi:10.1186/1471-2318-14-49.

Bourne, P., P. M. Camic, and S. J. Crutch. 2021. "Psychosocial outcomes of dyadic arts interventions for people with a dementia and their informal caregivers: A systematic review." *Health & Social Care in the Community* 29 (6):1632-1649. doi: doi:<https://dx.doi.org/10.1111/hsc.13267>.

Braspenning, A. M., K. Cranen, Ljae Snaphaan, and E. J. M. Wouters. 2022. "A Multiple Stakeholder Perspective on the Drivers and Barriers for the Implementation of Lifestyle Monitoring Using Infrared Sensors to Record Movements for Vulnerable Older Adults Living Alone at Home: A Qualitative Study." *International Journal of Environmental Research & Public Health [Electronic Resource]* 19 (1):570. doi:<https://dx.doi.org/10.3390/ijerph19010570>.

Brims, Lucy, and Kathryn Oliver. 2018. "Effectiveness of assistive technology in improving the safety of people with dementia: a systematic review and meta-analysis." *Aging and Mental Health*.23 (8):942-951: doi:10.1080/13607863.2018.1455805.

Brown, N., T. Cedar, and C. Tziraki. 2022. "Psychodrama with persons with dementia on zoom: Proof of concept." *Dementia* 21 (4):1289-1303. doi:<https://dx.doi.org/10.1177/14713012221074484>.

Camic, Paul M., Victoria Tischler, and Chantal Helen Pearman. 2014. "Viewing and making art together: A multi-session art-gallery-based intervention for people with dementia and their carers." *Aging & Mental Health* 18 (2):161-168. doi:<https://doi.org/10.1080/13607863.2013.818101>.

Campbell, N. L., M. A. Boustani, E. N. Skopelja, S. Gao, F. W. Unverzagt, and M. D. Murray. 2012. "Medication adherence in older adults with cognitive impairment: a systematic evidence-based review." *American Journal of Geriatric Pharmacotherapy* 10 (3):165-77. doi:<https://dx.doi.org/10.1016/j.amjopharm.2012.04.004>.

Cheng, Z., M. Zhou, and K. Sabran. 2024. "Mobile app-based interventions to improve the well-being of people with dementia: a systematic literature review." *Assistive Technology* 36 (1):64-74. doi:<https://dx.doi.org/10.1080/10400435.2023.2206439>.

Clark, P. A., and al et. 2004. "Outcomes for patients with dementia from the Cleveland Alzheimer's Managed Care Demonstration." *Aging and Mental Health* 8 (1):40-51.

Costa, Jan, and Kevin Doughty. 2009. "The role of reminder aids and systems to support independence in people with memory problems." *Journal of Assistive Technologies* 3 (2):60-64. doi:.

Curelaru, Aurora, Sarah J. Marzolf, Jean-Claude K. G. Provost, and Helen H. H. Zeon. 2021. "Social Isolation in Dementia: The Effects of COVID-19." *Journal for Nurse Practitioners* 17 (8):950-953. doi:10.1016/j.nurpra.2021.05.002.

de las Heras, Alejandro Sánchez-Rico, and Marta Burgos. 2017. "ICT4Life Integrated Care Platform." *International Journal of Integrated Care (IJIC)* 17:1-2. doi:10.5334/ijic.3477.

DePalma, Judith A. 2007. "Older Adults Dementia Care and Assessment Evidence." *Home Health Care Management & Practice* 19 (2):132-133. doi:<https://doi.org/10.1177/1084822306294705>.

Derbring, Sandra, Melissa Barbos Nordström, Jenny-Ann Svenningsson, Anna Ekström, Elias Ingebrand, Christina Samuelsson, Katja Laakso, and Margret Buchholz. 2023. "Effects of a digital reminiscing intervention on people with dementia and their care-givers and relatives." *Ageing & Society* 43 (9):1983-2000. doi:10.1017/S0144686X21001446.

Diaz Narvaez, E., and N. Rughwani. 2019. "Intergenerational programs: A way to improve quality of life in patients with dementia." *Journal of the American Geriatrics Society* 67:S93. doi:<https://dx.doi.org/10.1111/jgs.15898>.

Domenicucci, R., F. Ferrandes, M. Sarlo, E. Borella, and C. Belacchi. 2022. "Efficacy of ICT-based interventions in improving psychological outcomes among older adults with MCI and dementia: A systematic review and meta-analysis." *Ageing Research Reviews* 82:101781. doi:<https://dx.doi.org/10.1016/j.arr.2022.101781>.

Dulaney, S., K. Lui, J. Merrilees, A. Kuo, J. Choi, M. Ragosta, A. McNamara, J. Gurley, and K. L. Possin. 2019. "Care Ecosystem: Care Navigation for People Living Alone with Cognitive Impairment." *Alzheimer's and Dementia* 15:P1154. doi:<https://dx.doi.org/10.1016/j.jalz.2019.06.3521>.

Elliott, Melanie, and Paula Gardner. 2018. "The role of music in the lives of older adults with dementia ageing in place: a scoping review." *Dementia: the International Journal of Social Research and Practice* 17 (2):199-213. doi:10.1177/1471301216639424.

Enshaeifar, S., A. Zoha, A. Markides, S. Skillman, S. T. Acton, T. Elsaleh, M. Hassanpour, A. Ahrabian, M. Kenny, S. Klein, H. Rostill, R. Nilforooshan, and P. Barnaghi. 2018. "Health management and pattern analysis of daily living activities of people with dementia using in-home sensors and machine learning techniques." *PLoS ONE [Electronic Resource]* 13 (5):e0195605. doi:<https://dx.doi.org/10.1371/journal.pone.0195605>.

Femiola, Clementine, and Mary Tilki. 2017. "Dementia peer support: service delivery for the people, by the people." *Working with Older People: Community Care Policy & Practice* 21 (4):243-250. doi:10.1108/WWOP-08-2017-0020.

Ferreira-Brito, F., F. Ribeiro, D. Aguiar de Sousa, J. Costa, C. Caneiras, L. Carrico, and A. Verdelho. 2021. "Are Video Games Effective to Promote Cognition and Everyday Functional Capacity in Mild Cognitive Impairment/Dementia Patients? A Meta-Analysis of Randomized Controlled Trials." *Journal of Alzheimer's Disease* 84 (1):329-341. doi:<https://dx.doi.org/10.3233/JAD-210545>.

Foundations. 2014. *Housing health and care integration toolkit*. Glossop: Foundations.

Franco, G. C., F. Gallay, M. Berenguer, C. Mourrain, and P. Couturier. 2008. "Non-invasive monitoring of the activities of daily living of elderly people at home--a pilot study of the usage of domestic appliances." *Journal of Telemedicine & Telecare* 14 (5):231-5. doi:<https://dx.doi.org/10.1258/jtt.2008.071207>.

Garrido, S., E. Oliver, L. Pendergast, and A. Short. 2024. "Online music listening programs for older adults with dementia during the COVID-19 Pandemic: A feasibility study." *Arts in Psychotherapy* 87. doi:<https://dx.doi.org/10.1016/j.aip.2023.102097>.

Gerritzen, E. V., M. J. Hull, H. Verbeek, A. E. Smith, and B. de Boer. 2020. "Successful Elements of Intergenerational Dementia Programs: A Scoping Review: Research." *Journal of Intergenerational Relationships* 18 (2):214-245. doi:10.1080/15350770.2019.1670770.

Goodman-Casanova, J. M., E. Dura-Perez, J. Guzman-Parra, A. Cuesta-Vargas, and F. Mayoral-Cleries. 2020. "Telehealth Home Support During COVID-19 Confinement for Community-Dwelling Older Adults With Mild Cognitive Impairment or Mild Dementia: Survey Study." *Journal of Medical Internet Research* 22 (5):e19434. doi:<https://dx.doi.org/10.2196/19434>.

Greene, B., M. Ostroushko, and J. Melius. 2022. "Finding Joy and Purpose Through Singing: Giving Voice to People Living With Dementia." *Creative Nursing* 28 (4):274-278. doi:<https://dx.doi.org/10.1891/CN-2022-0048>.

Hagen, I., T. Holthe, J. Gilliard, P. Topo, S. Cahill, E. Begley, K. Jones, P. Duff, J. Macijauskiene, A. Budraitiene, S. Bjørneby, and K. Engedal. 2004. "Development of a protocol for the assessment of assistive aids for people with dementia." *Dementia (14713012)* 3 (3):281-296. doi:10.1177/1471301204045161.

Harris, P. B., and C. A. Caporella. 2014. "An intergenerational choir formed to lessen Alzheimer's disease stigma in college students and decrease the social isolation of people with Alzheimer's disease and their family members: a pilot study." *American Journal of Alzheimer's Disease & Other Dementias* 29 (3):270-81. doi:<https://dx.doi.org/10.1177/1533317513517044>.

Heins, P., L. M. M. Boots, W. Q. Koh, A. Neven, F. R. J. Verhey, and M. E. de Vugt. 2021. "The effects of technological interventions on social participation of community-dwelling older adults with and without dementia: A systematic review." *Journal of Clinical Medicine* 10.(11):2308: doi:<https://dx.doi.org/10.3390/jcm10112308>.

Holloway, J. C. 2022. "A Virtual Intervention for Isolation and Loneliness of Older Adults." *Journal of the American Geriatrics Society* 70:S42. doi:<https://dx.doi.org/10.1111/jgs.17755>.

Holthe, T., L. Halvorsrud, D. Karterud, K. A. Hoel, and A. Lund. 2018. "Usability and acceptability of technology for community-dwelling older adults with mild cognitive impairment and dementia: a systematic literature review." *Clinical Interventions In Aging* 13:863-886. doi:<https://dx.doi.org/10.2147/CIA.S154717>.

Holthe, T., L. Halvorsrud, and A. Lund. 2022. "Digital Assistive Technology to Support Everyday Living in Community-Dwelling Older Adults with Mild Cognitive Impairment and Dementia." *Clinical Interventions In Aging* 17:519-544. doi:<https://dx.doi.org/10.2147/CIA.S357860>.

Hum, A., R. Y. Tay, Y. K. Y. Wong, N. B. Ali, I. Y. O. Leong, H. Y. Wu, J. J. Chin, A. O. K. Lee, and M. Y. H. Koh. 2020. "Advanced dementia: an integrated homecare programme." *BMJ supportive & palliative care* 10 (4):e40. doi:<https://dx.doi.org/10.1136/bmjspcare-2019-001798>.

Innes, Anthea, Lorna Chesterton, Lydia Morris, Sarah K. Smith, and Sophie Bushell. 2021. "Perspectives of people living with dementia and their care partners about the impact on social health when participating in a co‐designed dementia café." *Health & Social Care in the Community*. *30*(4), e1375-e1383. doi:<https://doi.org/10.1111/hsc.13545>.

Inoue, Takenobu, Misato Nihei, Takuya Narita, Minoru Onoda, Rina Ishiwata, Ikuko Mamiya, Motoki Shino, Hiroaki Kojima, Shinichi Ohnaka, Yoshihiro Fujita, and Minoru Kamata. 2012. "Field-based development of an information support robot for persons with dementia." *Technology & Disability* 24 (4):263-271.

Irazoki, E., L. M. Contreras-Somoza, J. M. Toribio-Guzman, C. Jenaro-Rio, H. van der Roest, and M. A. Franco-Martin. 2020. "Technologies for Cognitive Training and Cognitive Rehabilitation for People With Mild Cognitive Impairment and Dementia. A Systematic Review." *Frontiers in Psychology* 11:648. doi:<https://dx.doi.org/10.3389/fpsyg.2020.00648>.

Joshi, P., K. Hendrie, D. J. Jester, D. Dasarathy, H. Lavretsky, B. S. Ku, H. Leutwyler, J. Torous, D. V. Jeste, and R. R. Tampi. 2023. "Social connections as determinants of cognitive health and as targets for social interventions in persons with or at risk of Alzheimer's disease and related disorders: a scoping review." *International Psychogeriatrics*:1-27. doi:<https://dx.doi.org/10.1017/S1041610223000923>.

Kang, H. S., I. S. Koh, K. Makimoto, and M. Yamakawa. 2023. "Nurses' perception towards care robots and their work experience with socially assistive technology during COVID-19: A qualitative study." *Geriatric Nursing* 50:234-239. doi:<https://dx.doi.org/10.1016/j.gerinurse.2023.01.025>.

Karkou, V., S. Aithal, M. Richards, E. Hiley, and B. Meekums. 2023. "Dance movement therapy for dementia." *Cochrane Database of Systematic Reviews* 8:CD011022. doi: doi:<https://dx.doi.org/10.1002/14651858.CD011022.pub3>.

Karp, J. F., J. W. Shega, N. E. Morone, and D. K. Weiner. 2008. "Advances in understanding the mechanisms and management of persistent pain in older adults." *British Journal of Anaesthesia* 101 (1):111-20. doi:<https://dx.doi.org/10.1093/bja/aen090>.

Kelly, L., I. Richardson, and H. Moss. 2023. "Reducing rural isolation through music: telehealth music therapy for community dwelling people living with dementia and their family caregivers in rural Ireland." *Rural & Remote Health* 23 (1):8162-8612. doi:<https://dx.doi.org/10.22605/RRH8162>.

Keogh, F., M. Pierce, K. Neylon, P. Fleming, L. Carter, S. O'Neill, and E. O'Shea. 2018. "Supporting older people with complex needs through the intensive home care package initiative: Findings from a prospective cohort study." *Age and Ageing. Conference: 66th Annual and Scientific Meeting of the Irish Gerontological Society: Transforming Ageing Across Borders. Cavan Ireland* 47. doi:<https://dx.doi.org/10.1093/ageing/afy140.126>.

Khosla, Rajiv, Mei-Tai Chu, Seyed Mohammad Sadegh Khaksar, Khanh Nguyen, and Toyoaki Nishida. 2021. "Engagement and experience of older people with socially assistive robots in home care." *Assistive Technology* 33 (2):57-71. doi:10.1080/10400435.2019.1588805.

Kim, M. J., J. Y. Song, J. W. Jang, S. Y. Lee, J. H. Jhoo, G. H. Byeon, and Y. Kim. 2022. "Improving Medication Adherence in Isolated Patients With Cognitive Impairment Using Automated Telephone Reminders." *Dementia and Neurocognitive Disorders* 21 (4):117-125. doi:<https://dx.doi.org/10.12779/dnd.2022.21.4.117>.

Kitamura, T., S. Shiota, S. Jinkawa, M. Kitamura, and S. Hino. 2018. "Effect of preceding home-visit nursing on time to discharge in hospitalization for the treatment of behavioural and psychological symptoms of dementia among patients with limited familial care." *Psychogeriatrics:The Official Journal of the Japanese Psychogeriatric Society* 18 (1):36-41. doi:<https://dx.doi.org/10.1111/psyg.12282>.

Klemets, J., J. Maattala, and I. Hakala. 2019. "Integration of an in-home monitoring system into home care nurses' workflow: A case study." *International Journal of Medical Informatics* 123:29-36. doi:<https://dx.doi.org/10.1016/j.ijmedinf.2018.12.006>.

Koh, W. Q., F. X. H. Ang, and D. Casey. 2021. "Impacts of Low-cost Robotic Pets for Older Adults and People With Dementia: Scoping Review." *JMIR Rehabilitation And Assistive Technologies* 8 (1):e25340. doi:<https://dx.doi.org/10.2196/25340>.

Koh, W. Q., T. Vandemeulebroucke, C. Gastmans, R. Miranda, and L. Van den Block. 2023. "The ethics of pet robots in dementia care settings: Care professionals' and organisational leaders' ethical intuitions." *Frontiers in psychiatry Frontiers Research Foundation* 14:1052889. doi:<https://dx.doi.org/10.3389/fpsyt.2023.1052889>.

Kontos, Pia, and al et. 2021. "Dancing with dementia: exploring the embodied dimensions of creativity and social engagement." *Gerontologist* 61 (5):714-723. doi:10.1093/geront/gnaa129.

Koowattanataworn, P., N. E. Stolwijk, H. H. Nap, S. Ipakchian Askari, B. M. Hofstede, R. Bevilacqua, G. Amabili, A. Margaritini, C. C. Lin, C. J. Lin, H. F. Chieh, Y. T. Wong, and F. C. Su. 2022. "The co-design of an AI-driven healthy ageing eco-system: User requirements from dementia formal carers...International Society for Gerontechnology 13th World Conference, October 22-26, 2022, Daegu, South Korea." *Gerontechnology* 21:2-2. doi: doi:10.4017/gt.2022.21.s.587.2.sp4.

Lariviere, M., F. Poland, J. Woolham, S. Newman, and C. Fox. 2021. "Placing assistive technology and telecare in everyday practices of people with dementia and their caregivers: findings from an embedded ethnography of a national dementia trial." *BMC Geriatrics* 21 (1):121. doi:<https://dx.doi.org/10.1186/s12877-020-01896-y>.

Liang, X., J. A. Batsis, Y. Zhu, T. M. Driesse, R. M. Roth, D. Kotz, and B. MacWhinney. 2022. "Evaluating Voice-Assistant Commands for Dementia Detection." *Computer Speech & Language* 72: 01297 doi:<https://dx.doi.org/10.1016/j.csl.2021.101297>.

Lion, K., D. Szczesniak, S. Evans, E. Farina, D. Brooker, R. Chattat, F. Meiland, R. M. Droes, and J. Rymaszewska. 2021. "Can we reduce the stigmatisation experience with psychosocial interventions? An investigation of the meeting centre support programme impact on people with cognitive impairments." *European Psychiatry* 64:S137-S137. doi:<https://dx.doi.org/10.1192/j.eurpsy.2021.378>.

Lion, Katarzyna Małgorzata, Dorota Szcześniak, Katarzyna Bulińska, Justyna Mazurek, Shirley B. Evans, Simon C. Evans, Francesca Lea Saibene, Alessia d'Arma, Claudia Scorolli, Elisabetta Farina, Dawn Brooker, Rabih Chattat, Franka J. M. Meiland, Rose-Marie Dröes, and Joanna Rymaszewska. 2021. "Does the Meeting Centre Support Programme decrease the experience of stigmatisation among people with cognitive deficits?" *Aging & Mental Health* 25 (1):160-169. doi:10.1080/13607863.2019.1683815.

Livingston, Gill, Lynsey Kelly, Elanor Lewis-Holmes, Gianluca Baio, Stephen Morris, Nishma Patel, Rumana Z. Omar, Cornelius Katona, and Claudia Cooper. 2014. "A systematic review of the clinical effectiveness and cost-effectiveness of sensory, psychological and behavioural interventions for managing agitation in older adults with dementia." *Health Technology Assessment* 18 (8):1-226. doi:10.3310/hta18390.

Lloyd-Williams, Mari, and al et. 2020. "Activities delivered at home by family carers to maintain cognitive function in people with dementia socially isolating during COVID-19: evidence for non-technology based activities / interventions."

Löbe, Clara, and Hanan AboJabel. 2022. "Empowering people with dementia via using intelligent assistive technology: A scoping review." *Archives of Gerontology & Geriatrics* 101:104699 doi:10.1016/j.archger.2022.

Lord, Kathryn, Jules Beresford-Dent, Penny Rapaport, Alex Burton, Monica Leverton, Kate Walters, Iain Lang, Murna Downs, Jill Manthorpe, Sue Boex, Joy Jackson, Margaret Ogden, and Claudia Cooper. 2020. "Developing the New Interventions for independence in Dementia Study (NIDUS) theoretical model for supporting people to live well with dementia at home for longer: a systematic review of theoretical models and Randomised Controlled Trial evidence." *Social Psychiatry & Psychiatric Epidemiology* 55 (1):1-14. doi:10.1007/s00127-019-01784-w.

Luker, J. A., A. Worley, M. Stanley, J. Uy, A. M. Watt, and S. L. Hillier. 2019. "The evidence for services to avoid or delay residential aged care admission: a systematic review." *BMC Geriatrics* 19: 1-20. doi:<https://dx.doi.org/10.1186/s12877-019-1210-3>.

MacAndrew, Margaret, Deborah Brooks, and Elizabeth Beattie. 2019. "NonPharmacological interventions for managing wandering in the community: a narrative review of the evidence base." *Health and Social Care in the Community* 27 (2):306-319. doi:10.1111/hsc.12590.

Maffioletti, V. L. R., M. A. T. Baptista, B. Abranches, G. Koatz, V. M. Rodrigues, A. Deslandes, and M. C. N. Dourado. 2021. "Virtual day center for people with dementia and their caregivers during the COVID-19 pandemic." *Dementia & Neuropsychologia* 15 (4):440-447. doi:<https://dx.doi.org/10.1590/1980-57642021dn15-040003>.

Maggio, M. G., C. De Domenico, A. Manuli, D. Latella, A. Marra, G. La Rosa, S. Portaro, and R. S. Calabro. 2023. "Alzheimer cafe: toward bridging the gap between cure and care in patients with dementia." *International Journal of Neuroscience* 133 (9):1024-1030. doi:<https://dx.doi.org/10.1080/00207454.2022.2040024>.

Malmgren Fange, A., G. Carlsson, C. Chiatti, and C. Lethin. 2020. "Using sensor-based technology for safety and independence - the experiences of people with dementia and their families." *Scandinavian Journal of Caring Sciences* 34 (3):648-657. doi:<https://dx.doi.org/10.1111/scs.12766>.

Manteau-Rao, M., and R. W. Levenson. 2018. "Unmet Needs of Persons with Dementia Living Alone, and How Smart Home Technologies Can Help." *Alzheimer's and Dementia* 14:P188-P189. doi:<https://dx.doi.org/10.1016/j.jalz.2018.06.2023>.

McGilton, K. S., A. Davis, N. Mahomed, J. Flannery, S. Jaglal, C. Cott, G. Naglie, and E. Rochon. 2012. "An inpatient rehabilitation model of care targeting patients with cognitive impairment." *BMC Geriatrics* 12:1-11. doi:<https://dx.doi.org/10.1186/1471-2318-12-21>.

McGrattan, Mairead, Cristín Ryan, Heather Barry, and Carmel Hughes. 2017. "Interventions to Improve Medicines Management for People with Dementia: A Systematic Review." *Drugs & Aging* 34 (12):907-916. doi:10.1007/s40266-017-0505-3.

Mole, Louise, Bridie Kent, Rebecca Abbott, Chloë Wood, and Mary Hickson. 2018. "The nutritional care of people living with dementia at home: A scoping review." *Health & Social Care in the Community* 26 (4):e485-e496. doi:10.1111/hsc.12540.

Moniz Cook, E. D., K. Swift, I. James, R. Malouf, M. De Vugt, and F. Verhey. 2012. "Functional analysis-based interventions for challenging behaviour in dementia." *Cochrane Database of Systematic Reviews* (2):N.PAG-N.PAG. doi:.

Mourant, Andrew. 2014. "From the grassroots up." *Mental Health Today*:10-11. doi:

Moyle, W., U. Arnautovska, T. Ownsworth, and C. Jones. 2017. "Potential of telepresence robots to enhance social connectedness in older adults with dementia: an integrative review of feasibility." *International Psychogeriatrics* 29 (12):1951-1964. doi:<https://dx.doi.org/10.1017/S1041610217001776>.

Moyle, W., J. Murfield, and K. Lion. 2021. "The effectiveness of smart home technologies to support the health outcomes of community-dwelling older adults living with dementia: A scoping review." *International Journal of Medical Informatics* 153:104513. doi:<https://dx.doi.org/10.1016/j.ijmedinf.2021.104513>.

Mulvenna, M., A. Hutton, V. Coates, S. Martin, S. Todd, R. Bond, and A. Moorhead. 2017. "Views of Caregivers on the Ethics of Assistive Technology Used for Home Surveillance of People Living with Dementia." *Neuroethics* 10 (2):255-266. doi:<https://dx.doi.org/10.1007/s12152-017-9305-z>.

Neylon, K., F. Keogh, M. Pierce, and P. Fleming. 2017. "Intensive homecare packages; Are they feasible for supporting people with dementia living alone?" *Age and Ageing* 46: 13. doi:<https://dx.doi.org/10.1093/ageing/afx144.173>.

Nourhashemi, F., S. Gillette-Guyonnet, S. Andrieu, Y. Rolland, P. J. Ousset, and B. Vellas. 2008. "A randomized trial of the impact of a specific care plan in 1120 Alzheimer's patients (PLASA Study) over a two-year period: design and baseline data." *Journal of Nutrition, Health & Aging* 12 (4):263-71.doi:.

Nygård, L., C. Ryd, M. Issakainen, K. Shastri, S. Marashi, A. C. Nedlund, A. Mäki-Petäjä-Leinonen, P. Flora, J. Boger, and A. Astell. 2020. "MCI@work: The role of technology in work and everyday life as experienced by people with MCI or early stage dementia...International Society for Gerontechnology's (ISG) 12th World Conference of Gerontechnology, October 6-9, 2020 (Virtual)." *Gerontechnology* 19:32-32. doi:10.4017/gt.2020.19.S.70020.

Nygard, L., S. Starkhammar, and M. Lilja. 2008. "The provision of stove timers to individuals with cognitive impairment." *Scandinavian Journal of Occupational Therapy* 15 (1):4-12. doi:<https://dx.doi.org/10.1080/11038120601124240>.

O'Connor, D. W., D. Ames, B. Gardner, and M. King. 2009. "Psychosocial treatments of psychological symptoms in dementia: a systematic review of reports meeting quality standards." *International Psychogeriatrics* 21 (2):241-51. doi:<https://dx.doi.org/10.1017/S1041610208008223>.

Park, Juyoung, Keri J. Heilman, Marlysa Sullivan, Jayshree Surage, Hannah Levine, Lillian Hung, María Ortega, Lisa Ann Kirk Wiese, and Hyochol Ahn. 2022. "Remotely supervised home-based online chair yoga intervention for older adults with dementia: Feasibility study." *Complementary Therapies in Clinical Practice* 48: 101617doi:10.1016/j.ctcp.2022.101617.

Pierce, M., F. Keogh, K. Neylon, O. Cosgrove, J. Linehan, M. Manning, R. Maguire, J. McCormack, B. Hannon, and K. Jordan. 2017. "Indicators on home based care for people with dementia: A tool for informing policy implementation and planning." *Age and Ageing* 46 doi:<https://dx.doi.org/10.1093/ageing/afx145.46>.

Pinto-Bruno, A. C., J. A. Garcia-Casal, E. Csipke, C. Jenaro-Rio, and M. Franco-Martin. 2017. "ICT-based applications to improve social health and social participation in older adults with dementia. A systematic literature review." *Aging & Mental Health* 21 (1):58-65. doi:<https://dx.doi.org/10.1080/13607863.2016.1262818>.

Price, C. 2007. "Monitoring people with dementia -- controlling or liberating?" *Quality in Ageing* 8 (3):41-44. doi: doi:10.1108/14717794200700020.

Prick, Anna-Eva, Jacomine de Lange, Erik Scherder, and Anne Margriet Pot. 2011. "Home-based exercise and support programme for people with dementia and their caregivers: study protocol of a randomised controlled trial." *BMC Public Health* 11 (1):894-894. doi: 10.1186/1471-2458-11-894.

Quail, Z., L. Bolton, and K. Massey. 2021. "Digital delivery of non-pharmacological intervention programmes for people living with dementia during the COVID-19 pandemic." *BMJ Case Reports* 14 (6):17.doi:<https://dx.doi.org/10.1136/bcr-2021-242550>.

Radke, A., W. Hoffmann, and B. Michalowsky. 2019. "Nd4 Who Benefits Most from Collaborative Dementia Care? A Post-Hoc Subgroup Cost-Effectiveness Analysis." *Value in Health* 22:S412. doi:<https://dx.doi.org/10.1016/j.jval.2019.09.080>.

Radke, A., B. Michalowsky, J. R. Thyrian, T. Eichler, F. Xie, and W. Hoffmann. 2020. "Who Benefits Most from Collaborative Dementia Care from a Patient and Payer Perspective? A Subgroup Cost-Effectiveness Analysis." *Journal of Alzheimer's Disease* 74 (2):449-462. doi:<https://dx.doi.org/10.3233/JAD-190578>.

Rahja, M., T. Comans, L. Clemson, M. Crotty, and K. Laver. 2018. "Are there missed opportunities for occupational therapy for people with dementia? An audit of practice in Australia." *Australian Occupational Therapy Journal* 65 (6):565-574. doi:<https://dx.doi.org/10.1111/1440-1630.12514>.

Rai, H. K., D. Kernaghan, L. Schoonmade, K. J. Egan, and A. M. Pot. 2022. "Digital Technologies to Prevent Social Isolation and Loneliness in Dementia: A Systematic Review." *Journal of Alzheimer's Disease* 90 (2):513-528. doi:<https://dx.doi.org/10.3233/JAD-220438>.

Research, National Institute For Health Research School for Social Care. 2015. *Best practice in social care and support for adults with concurrent sight loss and dementia within different housing settings*. London: NIHR School for Social Care Research.

Ridder, H. M., and D. Aldridge. 2005. "Individual music therapy with persons with frontotemporal dementia: singing dialogue." *Nordic Journal of Music Therapy* 14 (2):91-106. doi:10.1080/08098130509478132.

Rokstad, A. M. M., K. Engedal, O. Kirkevold, J. S. Benth, and G. Selbaek. 2018. "The impact of attending day care designed for home-dwelling people with dementia on nursing home admission: a 24-month controlled study." *BMC Health Services Research* 18:1-11. doi:<https://dx.doi.org/10.1186/s12913-018-3686-5>.

Russell, J., L. Hovey, and C. Fairlie. 2005. "Home care service for people with dementia: case study for the housing learning and improvement network." *Housing, Care & Support* 8 (3):17-21. doi: doi:10.1108/14608790200500020.

Salva, A., S. Andrieu, E. Fernandez, E. J. Schiffrin, J. Moulin, B. Decarli, Y. Guigoz, and B. Vellas. 2009. "Health and nutritional promotion program for patients with dementia (NutriAlz Study): design and baseline data." *Journal of Nutrition, Health & Aging* 13 (6):529-37. doi:

Samtani, S., A. Stevens, and H. Brodaty. 2021. "Preserving and enhancing social health in neurocognitive disorders." *Current Opinion in Psychiatry* 34 (2):157-164. doi: doi:<https://dx.doi.org/10.1097/YCO.0000000000000683>.

Schneider, C., M. Nissen, T. Kowatsch, and R. Vinay. 2024. "Impact of digital assistive technologies on the quality of life for people with dementia: A scoping review." BMJ Open *14(2), e080545* doi:<https://dx.doi.org/10.1101/2023.10.02.23296434>.

Schwartz, L. B. (2012). *The importance of health-related quality of life in persons with cognitive impairment*. The Johns Hopkins University.Scott, Iona, Claudia Cooper, Monica Leverton, Alex Burton, Jules Beresford‐Dent, Kenneth Rockwood, Laurie Butler, Penny Rapaport, and Jules Beresford-Dent. 2019. "Effects of nonpharmacological interventions on functioning of people living with dementia at home: A systematic review of randomised controlled trials." *International Journal of Geriatric Psychiatry* 34 (10):1386-1402. doi:10.1002/gps.5127.

Semple, Amy, Elizabeth Willis, and Waal Hugo de. 2015. *Peer support for people with dementia: a social return on investment (SROI) study*. London: Health Innovation Network South London.

Shannon, Kay, Kasia Bail, and Stephen Neville. 2019. "Dementia‐friendly community initiatives: An integrative review." *Journal of Clinical Nursing* 28 (11):2035-2045. doi:<https://doi.org/10.1111/jocn.14746>.

Sharew, N. T. 2022. "The Effect of Multimodal Non-pharmacological Interventions on Cognitive Function Improvement for People With Dementia: A Systematic Review." *Frontiers in Public Health* 10:894930. doi:<https://dx.doi.org/10.3389/fpubh.2022.894930>.

Sharew, N. T., S. C. Lam, T. D. Habtewold, and A. Y. M. Leung. 2021. "Effect of multimodal non-pharmacologic interventions on cognitive function for people with dementia: Systematic review." *Alzheimer's & dementia : the journal of the Alzheimer's Association* 17:e057657. doi:<https://dx.doi.org/10.1002/alz.057657>.

Shin, M. H., J. McLaren, A. Ramsey, J. L. Sullivan, and L. Moo. 2022. "Improving a Mobile Telepresence Robot for People With Alzheimer Disease and Related Dementias: Semistructured Interviews With Stakeholders." *24058297* 5 (2):e32322. doi:<https://dx.doi.org/10.2196/32322>.

Sixsmith, A. 2006. "New technologies support independent living and quality of life for people with dementia." *Alzheimer's Care Quarterly* 7 (3):194-202. doi: doi:.

Smith, S. K., and A. J. Astell. 2017. "Independent Living Functions for the Elderly (IN-LIFE): Supporting Communication in Dementia." *Studies in Health Technology & Informatics* 242:16-22. doi:

Souza, A. M. (2016). *Early stage memory loss interventions: utilization, impact, and the experience of living alone* (Doctoral dissertation).Starns, M. K., T. X. Karner, and R. J. Montgomery. 2002. "Exemplars of successful Alzheimer's demonstration projects." *Home Health Care Services Quarterly* 21 (3):141-75. doi:

Streater, Amy, Lauren Yates, Martin Orrell, Joe Rosen, Andy Taylor Smith, and Justine Schneider. 2020. "Interacting with television in one's own home: The development of a cognitive stimulation television pilot episode for older people with dementia (Innovative Practice)." *Dementia (14713012)* 19 (8):2881-2888. doi: 10.1177/1471301219836001.

Struckmeyer, L. R., and N. D. Pickens. 2016. "Home Modifications for People With Alzheimer's Disease: A Scoping Review." *The American journal of occupational therapy : official publication of the American Occupational Therapy Association* 70:p1-p9. doi: <https://dx.doi.org/10.5014/ajot.2015.016089>.

Sun, W. 2018. "Living Well with Dementia: The Role of Community-Based Programs in Promoting Social Connectedness for Persons with Dementia and Their Caregivers." *Alzheimer's and Dementia* 14:P947. doi:<https://dx.doi.org/10.1016/j.jalz.2018.06.1243>.

Sun, Winnie, Rabia Akhter, and Glory Gabel. 2022. "Developing and Evaluating Virtual Programs for Persons with Dementia During the Pandemic and Beyond...22nd International Conference on Integrated Care, May 23-25, 2022, Odense, Denmark." *International Journal of Integrated Care (IJIC)* 22:1-2. doi:10.5334/ijic.ICIC22358.

Thomas, Kali S., Jen Bunker, Emily Gadbois, Michelle Hilgeman, Ellen McCreedy, Whitney Mills, Katherine A. Ornstein, Jennifer Reckrey, and Roee Gutman. 2023. "Home-Delivered Meals and Nursing Home Placement Among People With Self-Reported Dementia: A Pilot Pragmatic Clinical Trial." *JAMA Network Open* 6 (12):e2347195-e2347195. doi: 10.1001/jamanetworkopen.2023.47195.

Tsuda, S., H. Inagaki, T. Okamura, M. Sugiyama, M. Ogawa, F. Miyamae, A. Edahiro, C. Ura, N. Sakuma, and S. Awata. 2022. "Promoting cultural change towards dementia friendly communities: a multi-level intervention in Japan." *BMC Geriatrics* 22 (1):360.doi:<https://dx.doi.org/10.1186/s12877-022-03030-6>.

Tulliani, N., M. Bissett, P. Fahey, R. Bye, and K. P. Y. Liu. 2022. "Efficacy of cognitive remediation on activities of daily living in individuals with mild cognitive impairment or early-stage dementia: a systematic review and meta-analysis." *Systematic Reviews* 11 (1):156. doi:<https://dx.doi.org/10.1186/s13643-022-02032-0>.

Van Assche, M., M. Petrovic, D. Cambier, P. Calders, P. Van Gelder, and D. Van de Velde. 2024. "The perspectives of older adults with mild cognitive impairment and their caregivers on the use of socially assistive robots in healthcare: exploring factors that influence attitude in a pre-implementation stage." *Disability & Rehabilitation Assistive Technology* 19 (1):222-232. doi:<https://dx.doi.org/10.1080/17483107.2022.2075477>.

van der Roest, H. G., F. J. Meiland, T. Haaker, E. Reitsma, H. Wils, C. Jonker, and R. M. Droes. 2008. "Finding the service you need: human centered design of a Digital Interactive Social Chart in DEMentia care (DEM-DISC)." *Studies in Health Technology & Informatics* 137:210-24. doi:.

van Leersum, C. M., K. E. Konrad, E. Siebrand, Z. B. Malik, M. E. M. den Ouden, and M. Bults. 2023. "Engaging older adults with a migration background to explore the usage of digital technologies in coping with dementia." *Frontiers in Public Health* 11:1125834. doi:<https://dx.doi.org/10.3389/fpubh.2023.1125834>.

Van Mierlo, L. D., H. G. Van der Roest, F. J. Meiland, and R. M. Droes. 2010. "Personalized dementia care: proven effectiveness of psychosocial interventions in subgroups." *Ageing Research Reviews* 9 (2):163-83. doi:<https://dx.doi.org/10.1016/j.arr.2009.09.002>.

Villars, Hélène, Charlotte Dupuy, Pauline Soler, Virginie Gardette, Maria E. Soto, Sophie Gillette, Fati Nourhashemi, and Brunovellas. 2013. "A follow-up intervention in severely demented patients after discharge from a special Alzheimer acute care unit: impact on early emergency room re-hospitalization rate." *International Journal of Geriatric Psychiatry* 28 (11):1131-1140. doi: doi:10.1002/gps.3932.

Waal, Hugo de. 2014. "Rethinking dementia: how autonomy and control can be fostered through the development of person centred services." *Working with Older People: Community Care Policy & Practice* 18 (2):82-89. doi:10.1108/WWOP-02-2014-0006.

Watari, K., J. L. Wetherell, M. Gatz, J. Delaney, C. Ladd, and D. Cherry. 2006. "Long distance caregivers: characteristics, service needs, and use of a long distance caregiver program." *Clinical Gerontologist* 29 (4):61-77. doi:10.1300/j018v29n04_05.

Waugh, F. 2009. "Where does risk feature in community care practice with older people with dementia who live alone?" *Dementia (14713012)* 8 (2):205-222. doi:10.1177/1471301209103255.

Wells, M. D., A. Morse, J. Barter, K. Mammino, A. A. Bay, T. Prusin, and M. E. Hackney. 2023. "Walk with Me Hybrid Virtual/In-Person Walking for Older Adults with Neurodegenerative Disease." *Journal of Visualized Experiments* 196 (6):16. doi:<https://dx.doi.org/10.3791/62869>.

Wenborn, J., G. Mountain, E. Moniz-Cook, F. Poland, M. King, R. Omar, A. O'Keeffe, S. Morris, E. Pizzo, S. Michie, M. Vernooij-Dassen, M. Graff, J. Hill, D. Challis, I. Russell, C. Sackley, S. Hynes, N. Crellin, J. Mundy, J. Burgess, T. Swinson, L. Di Bona, B. Field, C. Hart, J. Stansfeld, H. Walton, S. Rooks, R. Ledgerd, and M. Orrell. 2023. *National Institute for Health and Care Research. Programme Grants for Applied Research2023* 6:06. doi:<https://dx.doi.org/10.3310/RGTJ7429>.

Wenborn, Jennifer, Aidan G. O'Keeffe, Gail Mountain, Esme Moniz-Cook, Michael King, Rumana Z. Omar, Jacqueline Mundy, Jane Burgess, Fiona Poland, Stephen Morris, Elena Pizzo, Myrra Vernooij-Dassen, David Challis, Susan Michie, Ian Russell, Catherine Sackley, Maud Graff, Tom Swinson, Nadia Crellin, and Sinéad Hynes. 2021. "Community Occupational Therapy for people with dementia and family carers (COTiD-UK) versus treatment as usual (Valuing Active Life in Dementia [VALID]) study: A single-blind, randomised controlled trial." *PLoS Medicine* 18 (1):1-19. doi:10.1371/journal.pmed.1003433.

White, F. 2016. "Minds in motion: A community-based physical and social recreation program promoting a healthyactive lifestyle for people with dementia and their care partners." *Alzheimer's and Dementia* 12:P797-P798. doi:

Wilson, G. 2015. "WA4 Volunteering in partnership: a public health approach to delivering compassionate care to those at end of life and the frail elderly." *BMJ supportive & palliative care* 5:A2. doi:<https://dx.doi.org/10.1136/bmjspcare-2015-000906.4>.

Windischbauer, D., H. Eckardt, M. Morgenstern, K. Stoffel, and M. Clauss. 2023. "118 periprosthetic Vancouver B2 fractures operated with either open reduction and internal fixation or revision arthroplasty. An analysis of complications." *Swiss Medical Weekly* 153:55S. doi:

Wrede, C., A. Braakman-Jansen, and L. van Gemert-Pijnen. 2021. "Requirements for Unobtrusive Monitoring to Support Home-Based Dementia Care: Qualitative Study Among Formal and Informal Caregivers." *24058297* 4 (2):e26875. doi:<https://dx.doi.org/10.2196/26875>.

Wu, C. Y., K. Yu, S. E. Arnold, S. Das, and H. H. Dodge. 2023. "Application of the Personalized Medicine Approach to a Behavioral Intervention Study: The Internet-Based Conversational Engagement Clinical Trial (I-Conect)." *Journal of Prevention of Alzheimer's Disease* 10:S69. doi:<https://dx.doi.org/10.14283/jpad.2022.130>.

Wubbeler, M., J. R. Thyrian, B. Michalowsky, J. Hertel, F. Laporte Uribe, K. Wolf-Ostermann, S. Schafer-Walkmann, and W. Hoffmann. 2015. "Nonpharmacological therapies and provision of aids in outpatient dementia networks in Germany: utilization rates and associated factors." *Journal of multidisciplinary healthcare* 8:229-36. doi:<https://dx.doi.org/10.2147/JMDH.S80560>.

Xu, L., N. L. Fields, K. M. Daniel, D. J. Cipher, and B. A. Troutman. 2023. "Reminiscence and Digital Storytelling to Improve the Social and Emotional Well-Being of Older Adults With Alzheimer's Disease and Related Dementias: Protocol for a Mixed Methods Study Design and a Randomized Controlled Trial." *JMIR Research Protocols* 12:e49752. doi: <https://dx.doi.org/10.2196/49752>.

Zhu, X., M. He, Y. Dong, S. Zhang, S. Fang, W. Wang, M. Zhang, and J. Sun. 2023. "How tablets/applications enhance social connections and social support in people with dementia: A qualitative systematic review." *International Journal of Mental Health Nursing* 32 (3):727-743. doi::<https://dx.doi.org/10.1111/inm.13112>.

Zwierenberg, Erik, Henk Herman, Dirk Lukkien, Lotte Cornelisse, Evelyn Finnema, Ate Dijkstra, Mariët Hagedoorn, and Robbert Sanderman. 2018. "A lifestyle monitoring system to support (in)formal caregivers of people with dementia: Analysis of users need, benefits, and concerns." *Gerontechnology* 17 (4):194-205. doi: 10.4017/gt.2018.17.4.001.00.
