# Supplementary material for: Non‐Pharmacological Interventions for People With Dementia Who Live Alone: A Systematic Review
Source: Int J Geriatr Psychiatry. 2025 Feb 26;40(3):e70059. doi: 10.1002/gps.70059 (PMC11864918; doi:10.1002/gps.70059)
Supplement: Supplementary file 3 — Supporting Information S3 [file GPS-40-e70059-s003.docx]

**Supporting information:** Search strings

**MEDLINE**

| **#** | **Query** |
| --- | --- |
| 1 | (Dementia or dement* or "lewy bod*" or alzheimer* or "frontotemporal dement*" or "Vascular dement*").ti,ab,kw. |
| 2 | exp Dementia/ |
| 3 | exp Dementia, Vascular/ |
| 4 | *Frontotemporal Dementia/ |
| 5 | 1 or 2 or 3 or 4 |
| 6 | ("liv* alone" or "liv* on their own" or "lone living").ti,ab,kw. |
| 7 | (("one person" or one-person or single) adj2 household*).ti,ab,kw. |
| 8 | ("lone dwell*" or "lone-dwell*" or "dwell* alone").ti,ab,kw. |
| 9 | ("independent* liv*" or "liv* independent*").ti,ab,kf. |
| 10 | ("living arrangement*" or "living situation*").ti,ab,kw. |
| 11 | ("solo liv* or liv* solo or solo dwell*" or "solo-dwell*").ti,ab,kw. |
| 12 | *Social Isolation/ |
| 13 | "social* isolat*".ti,ab,kw. |
| 14 | 6 or 7 or 8 or 9 or 10 or 11 or 12 or 13 |
| 15 | 5 and 14 |
| 16 | limit 15 to (english language and yr="2000 -Current") |
| 17 | 6 or 7 or 8 or 9 or 10 or 11 |
| 18 | 5 and 17 |
| 19 | limit 18 to (english language and yr="2000 -Current") |

**EMBASE**

| **#** | **Query** |
| --- | --- |
| 1 | (Dementia or dement* or "lewy bod*" or alzheimer* or "frontotemporal dement*" or "Vascular dement*").ti,ab,kw. |
| 2 | exp dementia/ |
| 3 | 1 or 2 |
| 4 | ("liv* alone" or "liv* on their own" or "lone living").ti,ab,kw. |
| 5 | (("one person" or one-person or single) adj2 household*).ti,ab,kw. |
| 6 | ("lone dwell*" or "lone-dwell*" or "dwell* alone").ti,ab,kw. |
| 7 | ("independent* liv*" or "liv* independent*").ti,ab,kf. |
| 8 | ("living arrangement*" or "living situation*").ti,ab,kw. |
| 9 | ("solo liv* or liv* solo or solo dwell*" or "solo-dwell*").ti,ab,kw. |
| 10 | *social isolation/ |
| 11 | "social* isolat*".ti,ab,kw. |
| 12 | 4 or 5 or 6 or 7 or 8 or 9 or 10 or 11 |
| 13 | 3 and 12 |
| 14 | limit 13 to (english language and yr="2000 -Current") |
| 15 | 4 or 5 or 6 or 7 or 8 or 9 |
| 16 | 3 and 15 |
| 17 | limit 16 to (english language and yr="2000 -Current") |

**CINAHL**

| **#** | **Query** |
| --- | --- |
| S13 | (S4 OR S5 OR S6 OR S7 OR S8 OR S9 OR S10 OR S11) AND (S3 AND S12) |
| S12 | S4 OR S5 OR S6 OR S7 OR S8 OR S9 OR S10 OR S11 |
| S11 | (MM "Social Isolation") |
| S10 | TI("social* isolat*") OR AB("social* isolat*") |
| S9 | TI ("solo liv* or “liv* solo” or “solo dwell* or “solo-dwell*”) OR AB ("solo liv* or “liv* solo” or “solo dwell* or “solo-dwell*”) |
| S8 | TI ("living arrangement*" or "living situation*") OR AB ("living arrangement*" or "living situation*"). |
| S7 | TI (“independent* liv*” or “liv* independent*”) OR AB (“independent* liv*” or “liv* independent*”) |
| S6 | TI (“lone dwell*” or “lone-dwell*” or “dwell* alone”) OR AB (“lone dwell*” or “lone-dwell*” or “dwell* alone”) |
| S5 | TI ("one person household" OR "one-person household" OR "single household*") OR AB ("one person household" OR "one-person household" OR "single household*") |
| S4 | TI (“liv* alone” or “liv* on their own” or “lone living”) OR AB (“liv* alone” or “liv* on their own” or “lone living”) |
| S3 | S1 OR S2 |
| S2 | (MH "Dementia+") |
| S1 | TI (Dementia or dement* OR "lewy bod*" OR alzheimer* OR "frontotemporal dement*" OR “Vascular dement*”) OR AB (Dementia or dement* OR "lewy bod*" OR alzheimer* OR "frontotemporal dement*" OR “Vascular dement*”) |

1. **PSYCHINFO**

((MAINSUBJECT.EXACT.EXPLODE("Dementia") OR (tiab(Dementia OR dement* OR "lewy bod*" OR alzheimer* OR "frontotemporal dement*" OR "Vascular dement*") OR if(Dementia OR dement* OR "lewy bod*" OR alzheimer* OR "frontotemporal dement*" OR "Vascular dement*")))

AND

((tiab("liv* alone" OR "liv* on their own" OR "lone living") OR if("liv* alone" OR "liv* on their own" OR "lone living")) OR (tiab("one person household" OR "one-person household" OR "single household") OR if("one person household" OR "one-person household" OR "single household")) OR (tiab("lone dwell* " OR " lone-dwell* " OR " dwell* alone") OR if("lone dwell* " OR " lone-dwell* " OR " dwell* alone")) OR (tiab("independent* liv*" OR "liv* independent") OR if("independent* liv*" OR "liv* independent")) OR (tiab("living arrangement*" OR "living situation") OR if("living arrangement*" OR "living situation")) OR (tiab("solo liv* or " liv* solo " or " solo dwell* OR "solo-dwell") OR if("solo liv* or " liv* solo " or " solo dwell* OR "solo-dwell")) OR MJMAINSUBJECT.EXACT("Living Alone") OR MJMAINSUBJECT.EXACT("Living Arrangement") OR tiab("social* isolat*") OR MJMAINSUBJECT.EXACT("Social Isolation"))) AND la.exact("English")

**SOCIAL CARE ONLINE**

"Dementia" OR "lewy bodies" or "lewy body" OR "alzheimer" or "alzheimers" OR "frontotemporal dementia" or "Vascular dementia"
AND
 "living alone" OR "live alone" OR "lives alone" OR "live on their own" OR "lives on their own" OR "living on their own" OR "lone living" OR “lived alone” OR “lived on their own”  OR "one person household" OR "single household"  OR  "dwells alone" OR "lone dweller" OR "solo dweller" OR "lives solo" OR "solo living" OR “dwelling alone” OR  "lives independently" or "living independently" OR "lived independently" OR "independent living” OR "living arrangement" OR "living arrangements" OR "living situation" OR "living situations"
